# Engineering hepatitis B virus core particles for targeting HER2 receptors *in vitro* and *in vivo*

**DOI:** 10.1016/j.biomaterials.2016.12.012

**Published:** 2017-03

**Authors:** Izzat Fahimuddin Bin Mohamed Suffian, Julie Tzu-Wen Wang, Naomi O. Hodgins, Rebecca Klippstein, Mitla Garcia-Maya, Paul Brown, Yuya Nishimura, Hamed Heidari, Sara Bals, Jane K. Sosabowski, Chiaki Ogino, Akihiko Kondo, Khuloud T. Al-Jamal

**Affiliations:** a Institute of Pharmaceutical Science, King's College London, Franklin-Wilkins Building, 150 Stamford Street, London SE1 9NH, UK; b Randall Division of Cell & Molecular Biophysics, King's College London, New Hunt's House, London SE1 1UL, UK; c Department of Chemical Science and Engineering, Graduate School of Engineering, Kobe University, 1-1 Rokkodai, Nada, Kobe 657-8501, Japan; d Electron Microscopy for Materials Science (EMAT), University of Antwerp, Groenenborgerlaan 171, B-2020 Antwerp, Belgium; e Centre for Molecular Oncology, Bart's Cancer Institute, Queen Mary University of London, London EC1M 6BQ, UK

**Keywords:** Virus-like particles, Hepatitis B virus core particles, Human epidermal growth factor receptor 2, Affibody, Active targeting

## Abstract

Hepatitis B Virus core (HBc) particles have been studied for their potential as drug delivery vehicles for cancer therapy. HBc particles are hollow nano-particles of 30–34 nm diameter and 7 nm thick envelopes, consisting of 180–240 units of 21 kDa core monomers. They have the capacity to assemble/dis-assemble in a controlled manner allowing encapsulation of various drugs and other biomolecules. Moreover, other functional motifs, i.e. receptors, receptor binding sequences, peptides and proteins can be expressed. This study focuses on the development of genetically modified HBc particles to specifically recognise and target human epidermal growth factor receptor-2 (HER2)-expressing cancer cells, *in vitro* and *in vivo*, for future cancer therapy. The non-specific binding capacity of wild type HBc particles was reduced by genetic deletion of the sequence encoding arginine-rich domains. A specific HER2-targeting was achieved by expressing the Z_HER2_ affibodies on the HBc particles surface. *In vitro* studies showed specific uptake of Z_HER2_-ΔHBc particles in HER2 expressing cancer cells. *In vivo* studies confirmed positive uptake of Z_HER2_-ΔHBc particles in HER2-expressing tumours, compared to non-targeted ΔHBc particles in intraperitoneal tumour-bearing mice models. The present results highlight the potential of these nanocarriers in targeting HER2-positive metastatic abdominal cancer following intra-peritoneal administration.

## 1 Introduction

The effectiveness of detecting and treating cancer has remained a challenge for many researchers [1]. Additionally, delivering anti-tumour drugs to achieve a therapeutic effect without causing severe systemic side effects has proven to be challenging [2]. To address these issues, many efforts have been put into developing specific targeted carriers that can deliver the desired cargo selectively to tumour sites.

Virus-like particles (VLPs) provide an alternative platform for cell-targeted therapeutic delivery. VLPs are attractive as they are self-assembled, uniform, have well-defined geometry and are able to be tailored at an amino acid level by genetic modification [3]. Moreover, they form a closed structure that defines an interior environment capable of housing therapeutic or imaging agents and an exterior surface capable of multivalent presentation of targeting moieties [4], [5]. Hepatitis B Virus core (HBc) particles, examples of VLPs, have attracted many researchers as promising nanocarriers for cancer therapeutic studies [6].

HBc particles are hollow nanoparticles, 30–34 nm in diameter with 7 nm thickness envelopes, consisting of 180–240 units of 21 kDa core monomers [7], [8]. They are capable of non-specific binding to various cell types *via* the action of positively-charged arginine-rich domain. However, the arginine-rich domain of the core protein is not critical for the particle assembly [7], [9], [10]. The major immunodominant region (MIR) of HBc particles, located at the 78–83 amino acids (aa), is able to express immunological epitopes [11]. It has been shown that other functional motifs i.e., receptors [12], proteins [13] and element recognising low molecular mass substrates [14], can be expressed by genetic modification to this region.

Recently, affibody molecules, a new class of affinity ligands derived from the Z-domain in the binding region of *Staphylococcus aureus* protein A [15], have been the focus of researchers as a viable alternative to antibodies. Among the types of affibodies, monomeric Z_HER2:342_ (hereafter Z_HER2_) can specifically bind the HER2 over-expressed on the cell membrane surface of breast cancer and ovarian cancer cells [16]. Due to this attractive property, Z_HER2_ affibody makes a suitable targeting moiety to facilitate HER2 targeting by the nanocarriers.

In this study, we focused on the development of genetically modified HBc particles to specifically recognise and target HER2-expressing cancer cells *in vitro* and *in vivo*, qualitatively and quantitatively, for nucleic acid delivery applications. HER2 targeting was achieved by expression of Z_HER2_ affibodies in the HBc monomer. HBc particles were prepared using the *E. coli* expression system. HBc particles morphology was confirmed by atomic force microscopy (AFM) and cryo transmission electron microscopy (Cryo-TEM). Protein specificity was confirmed by Western blotting. A range of cells expressing different levels of HER2 were treated with fluorescently labelled HBc particles and the cell uptake was assessed using flow cytometry. HBc particles were then radiolabelled with technetium-99m (^99m^Tc), using the previously reported hexahistidine sequence (His-tag) labelling protocol [17]. Single-photon emission computed tomography/computerised tomography (SPECT/CT) imaging and quantitative gamma counting were performed to characterise the organ biodistribution profile of the HER2 specific-targeting HBc particles in tumour-bearing mice.

## 2 Materials and methods

### 2.1 Materials

Please refer to Supporting information for the list of materials used.

### 2.2 Methods

#### 2.2.1 Expression, purification and assembly of HBc particles

*E. coli* BL21 (DE3) was transformed with plasmids for expression of wild type HBc, ΔHBc or Z_HER2_-ΔHBc cultured in 10 mL of Auto-Induction Media Terrific broth (AIM-TB) media in the presence of 100 μg/mL ampicillin and grown at 37 °C for 16 h using an incubator shaker. The culture was then diluted with 500 mL of fresh AIM-TB media in the presence of 100 μg/mL ampicillin and grown at 25 °C for 72 h. Cells were harvested at 5000 rpm, 4 °C for 15 min. Pelleted cells were re-suspended in the 30 ml of lysis buffer (50 mM Tris, 100 mM NaCl, 5 mM EDTA, 0.2% Triton X-100, 1x cOmplete™ protease inhibitor pH 8.0). The cells were treated with RNase A at final concentration 5 μg/mL at 4 °C for overnight. The lysate was sonicated using a probe sonicator on ice by three cycles for 1 min each with 1 min intervals to avoid heating the material. The supernatant was removed by centrifugation at 12,000 rpm, 4 °C for 30 min. The core particles in the cell pellet were washed in 30 ml of lysis buffer and collected by centrifugation at 12,000 rpm, 4 °C for 30 min. The cell pellet containing HBc, ΔHBc or Z_HER2_-ΔHBc particles was denatured in 40 ml of dissociation buffer (8 M urea, 200 mM NaCl, 50 mM sodium carbonate, 10 mM 2-mercaptoethanol, pH 9.5) by overnight incubation at 4 °C. Then, the pellet was discarded by centrifugation at 12,000 rpm, 4 °C for 30 min.

Soluble fraction containing-contaminating proteins were separated from HBc particle proteins using Ni^2+^-chelate affinity chromatography. A column with 6 mL of cOmplete™ His-Tag Purification Resin (Roche, Germany) was equilibrated with 3-times bed-volume (18 mL) of dissociation buffer. The column was loaded with the protein probe and washed with 18 mL of dissociation buffer. Bound HBc particle proteins were eluted with 14 mL of elution buffer (2 M urea, 200 mM NaCl, 50 mM sodium carbonate, 10 mM 2-mercaptoethanol, 1 M imidazole, pH 9.5). The eluted material was collected in 1 mL fractions. The aliquots of each fraction were subjected to sodium dodecyl sulphate-polyacrylamide gel electrophoresis (SDS-PAGE) and stained with Coomassie Brilliant Blue (CBB) to analyse their purity.

Fractions containing the HBc protein were re-assembled to particles by the removal of the urea. Specifically, protein fractions were dialysed against 2 L of dialysis buffer 1 (0.5 M urea, 100 mM Tris, 150 mM NaCl, 2 mM DTT, 1 mM EDTA, 10 mM CaCl_2_, pH 8.0) using SnakeSkin™ Dialysis Tubing, 10K MWCO (Thermo Scientific, USA) at 4 °C for 4 h, allowing HBc protein to start assembling. Then, the solution was dialysed against dialysis buffer 2 (100 mM Tris, 150 mM NaCl, 1 mM EDTA, 10 mM CaCl_2_, pH 8.0) to completely removed urea and DTT, to fully assemble the HBc particles. HBc particles were filtered using 0.44 μm filter to remove any aggregates. All protein concentrations were measured using NanoDrop™ ND-1000 UV–Vis Spectrophotometer (Thermo Fisher Scientific, USA).

#### 2.2.2 Atomic force microscopy (AFM) analysis of purified HBc particles

One hundred microliter of 10 μg/mL of purified wild type HBc, ΔHBc or Z_HER2_-ΔHBc particles was deposited on mica surfaces for 5 min and then flushed with air. Tapping mode AFM analysis (TM-AFM) on the mica substrates were carried out in air at 25 °C using a Bruker Dimension ICON with Scan Assist. The surfaces were imaged with a general purpose-tapping tip made by MikroMasch in Estonia (NSC15/no Al, tip radius < 10 nm; tip height = 20–25 μm; cone angle < 40°; cantilever thickness = 3.5–4.5 μm; cantilever width = 32 -28 μm; cantilever length = 120–130 μm; frequency *f*_*0*_ = 265–400 kHz; force constant k = 20–75 N m^−1^, VEECO, USA). The statistical analysis of the AFM images was carried out using WSxM v5.0 Developed 6.2 software (Spain).

#### 2.2.3 Cryo-transmission electron microscopy (Cryo-TEM) of HBc particles

The shape, morphology and size distribution of HBc particles was evaluated using low electron dose cryo-transmission electron microscopy (cryo-TEM). Cryo-TEM enables the investigation of colloidal dispersion [18] and biological origin samples like viruses [19] and proteins [20], close to their native state. A drop of solution (3 μl) was applied on hydrophilic TEM Quantifoil grids. The grids were then blotted for 2 s and plunged into the liquid ethane pool using a FEI Vitrobot mark IV, in order to produce a thin vitreous ice layer with embedded assemblies in the holes of the grid. Digital cryo-TEM images were acquired using a FEI Tecnai Spirit operated at 120 kV using a Gatan 626 cryo-transfer tomography holder.

#### 2.2.4 SDS-PAGE and western blot analysis of WT-HBc particles

The expression of each WT-HBc, ΔHBc or Z_HER2_-ΔHBc monomer was confirmed by western blotting. The purified WT-HBc, ΔHBc and Z_HER2_-ΔHBc particles were analysed by SDS-PAGE and electro-transferred onto a nitrocellulose membrane. For the detection of the His6-tag, rabbit anti-6-His antibody (Bethyl Laboratories, USA) was used as a primary antibody at 1:1000 dilution for immunoblotting, followed by HRP-linked anti-rabbit (Cell Signalling Technology, USA) at 1:1000 dilution and Precision Protein StrepTactin-HRP Conjugate (Bio-Rad Laboratories, USA) at 1:10,000 dilution for secondary antibody. For the detection of the His6-tag, mouse anti-HBc antibody (Merck Millipore, USA) was used as a primary antibody at 1:1000 dilution for immunoblotting, followed by HRP-linked anti-mouse (Cell Signalling Technology, USA) at 1:1000 dilution and Precision Protein StrepTactin-HRP Conjugate (Bio-Rad Laboratories, USA) at 1:10,000 dilution for secondary antibody. The specific bands were detected with enhanced chemiluminescence (ECL) detection system. The membrane was imaged using the ChemiDoc™MP (Bio-Rad Laboratories, USA) and analysed with Image Lab (Bio-Rad Laboratories, USA) software.

#### 2.2.5 Fluorescence labelling of HBc particles

Purified HBc particles were reacted with Alexa Fluor™ 488 Succinimidyl Esters (Invitrogen Life Technologies, USA) at 40:1 HBc:dye mass ratio in phosphate-buffered saline (PBS) buffer for 1 h at room temperature with stirring. The mixture was then dialysed using SnakeSkin™ Dialysis Tubing, 10K MWCO (Thermo Scientific, USA) against PBS buffer at 4 °C for overnight to remove free Alexa Fluor™ 488. To evaluate the fluorescence labelling efficiency, a standard curve of Alexa Fluor™ 488-labelled WT-HBc, ΔHBc or Z_HER2_- ΔHBc was prepared. Fluorescence intensity was measured at 485 nm and 520 nm excitation and emission wavelengths, respectively, at 25 °C using a BMG FLUOstar Omega fluorometer.

#### 2.2.6 Cell culture

A panel of cell lines, of varied degree of HER2 expression, was used in this study. The MDA-MB-435-MLE human melanoma (parent cell line: MDA-MB-435; ATCC, HTB-129) were cultured in DMEM media supplemented with 10% FBS, 50 U/mL penicillin, 50 μg/mL streptomycin, 1% L-glutamine, at 37 °C in 5% CO_2_. MDA-MB-435-MLE cells were obtained by transfecting the parent cell line MDA-MB-435 with MUC1, luciferase and ErbB2 genes as previously reported [21]. MDA-MB-435-MLE was a kind gift from Dr John Maher (Research Oncology Group, King's College London, UK). The MDA-MB-468 human breast adenocarcinoma (MDA-MB-468; ATCC, HTB-132), MDA-MB-231 human breast carcinoma (MDA-MB-231; ATCC, HTB-26), SKBR-3 human breast carcinoma (SKBR3; ATCC, HTB-30) and BT-474 human breast ductal carcinoma (BT-474; ATCC, HTB-20) were cultured in DMEM media supplemented with 10% FBS, 50 U/mL penicillin, 50 μg/mL streptomycin, 1% L-glutamine, at 37 °C in 5% CO_2_. All cells were routinely grown in 75 cm^2^ canted-neck tissue culture flasks and passaged twice a week using Trypsin/EDTA at 80% confluency.

#### 2.2.7 Uptake studies *in vitro* by flow cytometry

MDA-MB-468, MDA-MB-231, SKBR-3 and BT-474 cells were seeded at a density of 1 × 10^5^ cells/mL in 24-well plates and allowed to attach overnight. After washing with PBS buffer, cells were then treated with fluorescent Alexa Fluor™ 488-labelled HBc, ΔHBc or Z_HER2_-ΔHBc particles at increasing concentrations of 10, 20 or 40 μg/mL in serum-free media for 1, 4 or 24 h. For the 24 h treatment, after 4 h incubation, FBS was added to each well to reach 10% v/v final FBS concentration. Cells were re-incubated for another 20 h. After treatment, cells were washed twice with PBS buffer, trypsinised and centrifuged at 1500 rpm for 5 min and the cell pellet was re-suspended in 250 μl of PBS buffer. The internalisation of HBc particles was studied on 10,000 gated cells by detecting Alexa Fluor™ 488 fluorescence using FL-1 channel detector and BD FACS Calibur flow cytometer (BD Biosciences, USA). The measurements were done in triplicate and the data were analysed using BD CellQuest software (BD Biosciences, USA).

#### 2.2.8 Preparation of [^99m^Tc(CO_3_)]^+^

IsoLink kits (Mallinckrodt Medical BV, Petten, Netherlands) were reconstituted in 1 mL deionised water and subdivided into aliquots of 260 μL in microcentrifuge tubes. Aliquots were stored at −80 °C. Upon use, an aliquot of the reconstituted kits were allowed to thaw at room temperature, and were added to 1.5 GBq of 500 μL [^99m^TcCO_4_]- (St. Bartholomew's Hospital Radiopharmacy, London, UK) in glass vial and incubated for 30 min at 100 °C. The glass vial was then allowed to cool on ice and shortly centrifuged at room temperature to accumulate any condensed solution. The solution was then neutralised with 40 μL of 1 M HCl to pH approximately 7.0, giving a total of volume of 800 μl of [^99m^Tc(CO3)]^+^. A needle was inserted to vent the gas generated from the reaction.

#### 2.2.9 [^99m^Tc(CO3)]^+^ radiolabelling of HBc particles and serum stability

NaCl, at 5.5 M, was added to [^99m^Tc(CO_3_)]^+^ at a 1:1 vol ratio in a microcentrifuge tube to achieve a final [Na]^+^ salt concentration of 0.63 M at the end of the labelling reaction. For gamma counting studies, ΔHBc (1 mg/mL) or Z_HER2_-ΔHBc (1 mg/mL) particles in PBS buffer were radiolabelled by incubating 100 μl of each protein solution with up to 20 MBq in 20 μl of Na^+^-[^99m^Tc(CO3)]^+^. For SPECT/CT imaging, both HBc particles (2 mg/mL) were also radiolabelled with up to 75 MBq in 80 μl of Na^+^-[^99m^Tc(CO_3_)]^+^. The labelling reaction was carried out at 37°C and monitored for 2 h with labelling efficiency determined at 60 and 120 min using instant thin layer chromatography (iTLC) using citrate buffer as the mobile phase. This system provided a good separation between the [^99m^Tc(CO_3_)]^+^-labelled HBc particles and unbound [^99m^Tc(CO_3_)]^+^. Strips were allowed to dry before being developed and counted quantitatively using a cyclone phosphor detector (Packard Biosciences, UK). The ^99m^Tc-ΔHBc and ^99m^Tc-Z_HER2_-ΔHBc particles were buffer exchanged to PBS buffer using Vivaspin MWCO 10 kDa before being injected into animals. This also ensured that no unbound [^99m^Tc(CO_3_)]^+^ was present prior to injection. Solutions of purified ^99m^Tc-ΔHBc and ^99m^Tc-Z_HER2_-ΔHBc particles (∼200 μl per injection dose, 50 μg of protein) were collected and examined again by iTLC.

The stability of radiolabelled ^99m^Tc-ΔHBc and ^99m^Tc-Z_HER2_-ΔHBc particles was determined by incubating 5 μL of each sample with serum (5 μL) or PBS (5 μL) at 37 °C. After 24 h, 5 μL of the incubated samples were spotted to the iTLC paper strips, which were developed and quantified as described above. Unbound [^99m^Tc(CO_3_)]^+^ was detected at the solvent front while [^99m^Tc(CO_3_)]^+^-labelled HBc particles was retained at the solvent front.

#### 2.2.10 Animal studies and tumour inoculation

All animal experiments were performed in compliance with the UK Home Office (1989) Code of Practice for the housing and care of Animals used in Scientific Procedures.

For intraperitoneal model, the harvested MDA-MB-435-MLE cells were suspended in PBS buffer (pH 7.4) and injected intraperitoneally in the male NSG mice aged 4–6 weeks (Charles River, UK) at a concentration of 5.0 × 10^6^ cells in 100 μL. For the mammary fat pad (MFP) model, the harvested MDA-MB-435-MLE cells were suspended in PBS buffer (pH 7.4) and mixed with BD Matrigel™ (BD Bioscience, USA) at 1:1 ratio. Cell suspension was subcutaneously injected into the mammary fat pad of the female NSG mice aged 4–6 weeks (Charles River, UK) at a concentration of 5.0 × 10^5^ cells in 20 μL. For SPECT/CT imaging studies, both models were inoculated in the female SCID/Beige mice aged 4–6 weeks (Charles River, UK).

After inoculation, the tumour growth was analysed by bioluminescent imaging of mice every 7 days, from day 7 post-inoculation, using IVIS Lumina series III *In Vivo* Imaging Device (IVIS) (Perkin Elmer, the Netherlands). Mice were subcutaneously injected D-luciferin (150 mg of luciferin/kg) (Perkin Elmer, the Netherlands). Animals were imaged at 12 min post-injection and analysed using Living Image software (Perkin Elmer, the Netherlands). Photon flux from the tumour is proportional to the number of live cells expressing luciferase.

#### 2.2.11 Live small animal SPECT/CT imaging studies

ΔHBc and Z_HER2_-ΔHBc particles were radiolabelled with ^99m^Tc as described in previous sections. Whole body imaging of mice injected with ^99m^Tc-ΔHBc or ^99m^Tc-Z_HER2_-ΔHBc particles was carried out using SPECT/CT imaging. For the intraperitoneal model, MDA-MB-435-MLE tumour-bearing SCID/Beige mice were injected intraperitoneally in the right lower quadrant of the abdomen with 150 μl PBS solution containing 6–14 MBq ^99m^Tc-ΔHBc or ^99m^Tc-Z_HER2_-ΔHBc particles (corresponding to 200 μg protein/mouse). For the mammary fat pad model, MDA-MB-435-MLE tumour-bearing SCID/Beige mice were injected intratumourally with 10 μl PBS solution containing 4–9 MBq ^99m^Tc-ΔHBc or ^99m^Tc-Z_HER2_-ΔHBc particles (corresponding to 200 μg protein/mouse). SPECT/CT imaging was carried out at 0–30 min, 4 h and 24 h post-injection under anaesthesia with 1.5% isoflurane/98.5% oxygen using Nano-SPECT/CT Scanner (Bioscan, Washington DC, USA). SPECT acquisitions were obtained using 24 projections over 30 min using a four-head scanner with 1.4 mm pinhole collimators. CT scans were carried out after the SPECT acquisition. SPECT and CT images were analysed using InVivoScope™ software (Bioscan, Washington DC, USA).

#### 2.2.12 Quantitative biodistribution studies by gamma scintigraphy

Quantitative biodistribution studies of radiolabelled ^99m^Tc-HBc particles including organ biodistribution, blood circulation and excretion profile were performed in intraperitoneal or mammary fat pad models of MDA-MB-435-MLE tumour-bearing NSG mice using gamma. Mice were randomly divided into three groups of three mice each; [^99m^Tc(CO)_3_]^+^, ^99m^Tc-ΔHBc or ^99m^Tc-Z_HER2_-ΔHBc group. For the intraperitoneal model, mice were injected intraperitoneally *via* the right lower quadrant of the abdomen with 150 μL PBS solution containing 4–6 MBq ^99m^Tc-ΔHBc or ^99m^Tc-Z_HER2_-ΔHBc particles (corresponding to 50 μg protein/mouse). For the MFP model, mice were injected intratumourally with 150 μL or 10 μL PBS solution containing 4–6 MBq ^99m^Tc-ΔHBc or ^99m^Tc-Z_HER2_-ΔHBc (corresponding to 50 μg protein/mouse), respectively. Equivalent volumes and radioactivity of [^99m^Tc(CO)_3_]^+^ alone was used as a negative control. Tumours and other organs and tissues, *i.e.* skin, liver, spleen, heart, lung, muscle, bone (femur), brain, stomach, intestine, tail and carcass were removed, weighed and the radioactivity was measured by a gamma counter (1280 CompuGamma Universal Gamma Counter, LKB Wallac, Finland), using the appropriate windows for ^99m^Tc. Results were expressed as a percentage of injected dose per gram tissue (%ID/g of tissues) as means ± SD (n = 3).

## 3 Results

### 3.1 Plasmids construction

**Fig. 1:**
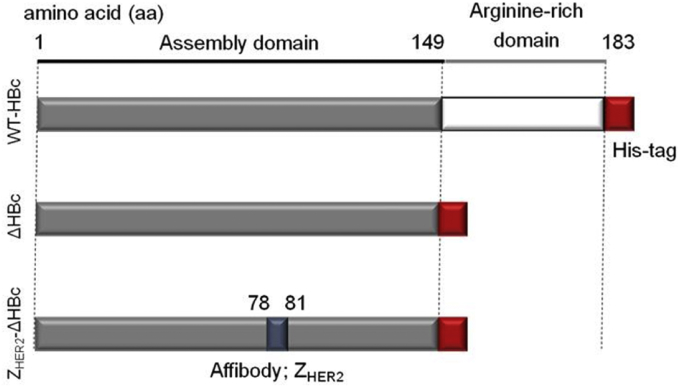
**Amino acid sequence of the wild type (WT) and recombinant HBc particles used in this study**. Wild-type HBc (WT-HBc) protein consists of an assembly domain (grey colour) and an arginine-rich domain (white colour). In the truncated HBc (ΔHBc) particles, the arginine-rich domain (150–183 aa) was deleted. In the HER2-targeting HBc (Z_HER2_-ΔHBc) particles, a Z_HER2_ affibody (blue colour) was inserted between 78 and 81 aa. For all constructs, His-tag (red colour) was fused to the C-termini to aid protein purification. (For interpretation of the references to colour in this figure legend, the reader is referred to the web version of this article.)

All plasmids depicted in Fig. 1 were constructed and prepared as described by Y. Nishimura et al. [22]. Plasmid expressing WT-HBc [22] with His6-tag-fused at the C-terminal was constructed. A full-length monomer consists of 183 aa residues (Fig. 1). A plasmid expressing truncated HBc (ΔHBc [22]) was constructed to reduce the non-specific binding capacity of WT-HBc particles, by deletion of the sequence encoding arginine-rich domains. Finally, a plasmid expressing Z_HER2_-ΔHBc [22] was constructed, by inserting a HER2 target-cell-specific affibody, Z_HER2_ between 78 and 81 aa sequences of the ΔHBc monomer to express the targeting moiety within HBc particles. All HBc plasmids were used to transform *E. coli*, and HBc proteins were expressed, purified and re-assembled as described in the ‘Methods’ section.

### 3.2 Protein yield and purity

The yield and characteristics of the various particles are shown in Table 1. A OD260/OD280 ratio greater than 0.6 indicates nucleic acid contamination in the protein samples [23].

### 3.3 Size and morphological examination of assembled HBc particles

The diameter of WT-HBc particles was in the range of 33.77 ± 4.58 nm obtained by atomic force microscopy (AFM) (Table 1). The removal of the arginine-rich domain sequence or the introduction of Z_HER2_ affibody did not lead to statistically significant differences in HBc particles' diameters (Table 1).

**Fig. 2:**
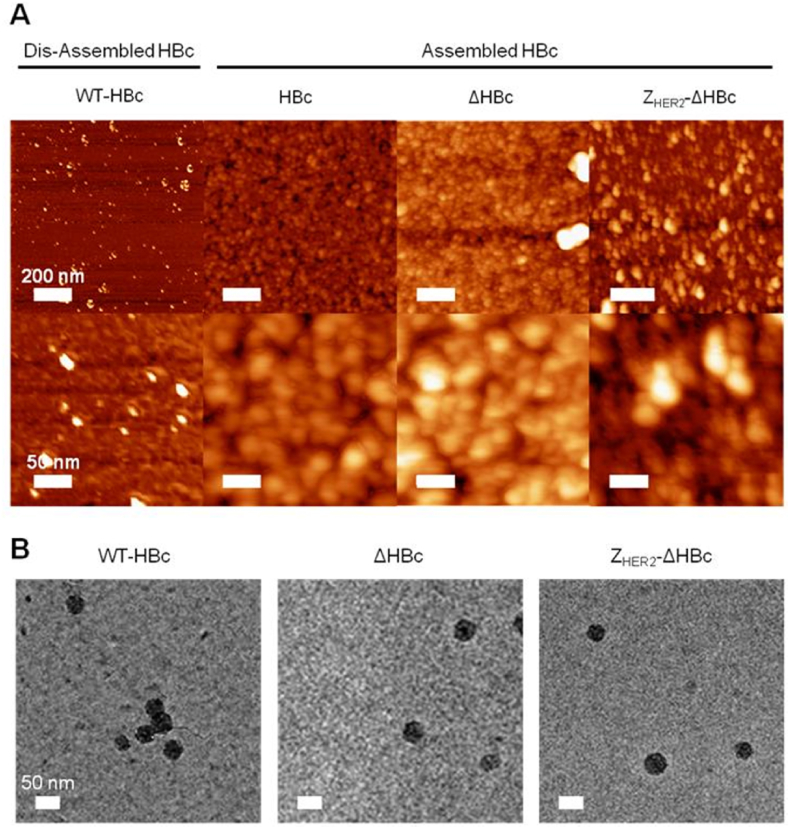
**Morphological analysis of purified HBc core particles with Atomic Force Microscopy (AFM) and Cryo-Transmission Electron Microscopy (Cryo-TEM). (A)** AFM images using tapping mode AFM (TM-AFM) and **(B)** cryo-EM images of HBc particles. Dis-assembled HBc were achieved by dilution in distilled water at 40 °C for 10 min. In A, HBc particles were deposited on the mica substrates and measurements were carried out in air at 25 °C using a Bruker Dimension ICON with Scan Assist. In B, 3 μL of 10 mg/mL assembled HBc particles solution was applied on hydrophilic TEM Quantifoil grids and plunged into the liquid ethane pool to produce a thin vitreous ice layer. Cryo-TEM images were acquired using a FEI Tecnai Spirit operated at 120 kV using a Gatan 626 cryo-transfer tomography holder. Dis-assembled HBc appeared as irregular structures while assembled HBc (WT-HBc, ΔHBc, and Z_HER2_-ΔHBc) particles appeared spherical in shape.

The morphology of assembled HBc particles was examined initially by AFM. As shown in Fig. 2A, HBc particles are spherical in shape and showed a homogenous size distribution. Expectedly, when HBc particles were dissociated by dilution in distilled water, the “dis-assembled” particles resulted in irregular and ‘broken’ structures, confirming that the addition of distilled water was an efficient method to dis-assemble these particles. No alteration in particle shape occurred with the deletion of arginine-rich domain (ΔHBc) or insertion of Z_HER2_ affibody (Z_HER2_-ΔHBc) Fig. 2A. The 2D bright-field projections from cryo-TEM suggested that all HBc particles, shown as electron-dense nanoparticles, are spherical in shape confirming the success of the self-assembly process (Fig. 2B).

### 3.4 Western blotting of HBc particles

**Fig. 3:**
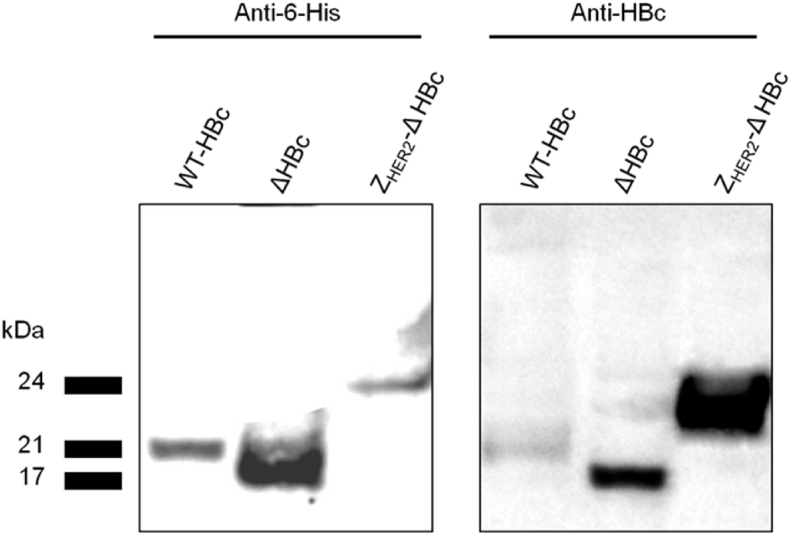
**Immuno-specificity of HBc particles by Western blotting**. Denatured HBc samples were subjected to SDS-PAGE followed by immune-blotting using anti-6-His and anti-HBc antibodies. Results confirmed the presence of specific protein bands at the expected molecular weight band; 21 kDa, 17 kDa and 24 kDa for WT-HBc, ΔHBc and ZHER2-ΔHBc, respectively.

Western blotting analysis was performed by immune-blotting using anti-6-His and anti-HBc antibodies to confirm the expression and purification of all HBc particle types. Results showed the presence of specific protein bands at the expected molecular weight bands using both antibodies at 21 kDa, 17 kDa and 24 kDa for HBc, ΔHBc and Z_HER2_-ΔHBc, respectively (Fig. 3). This confirms the successful expression and purification of HBc particles.

### 3.5 Intracellular uptake of HBc particles in cancer cells *in vitro*

HER2 expression levels in a range of cancer cell lines were examined using flow cytometry. Based on the “mean fluorescence intensity” (MFI) values, HER2 expression levels on cells were ranked in the following order: MDA-MB-468 < MDA-MB-231 < SKBR-3 < BT-474. Cells were classified as negative (−) (MDA-MB-468), low expressing (+) (MDA-MB-231) or over expressing (+++) (SKBR-3, BT-474) for HER2 receptors (See Supplementary Information, Fig. S1).

**Fig. 4:**
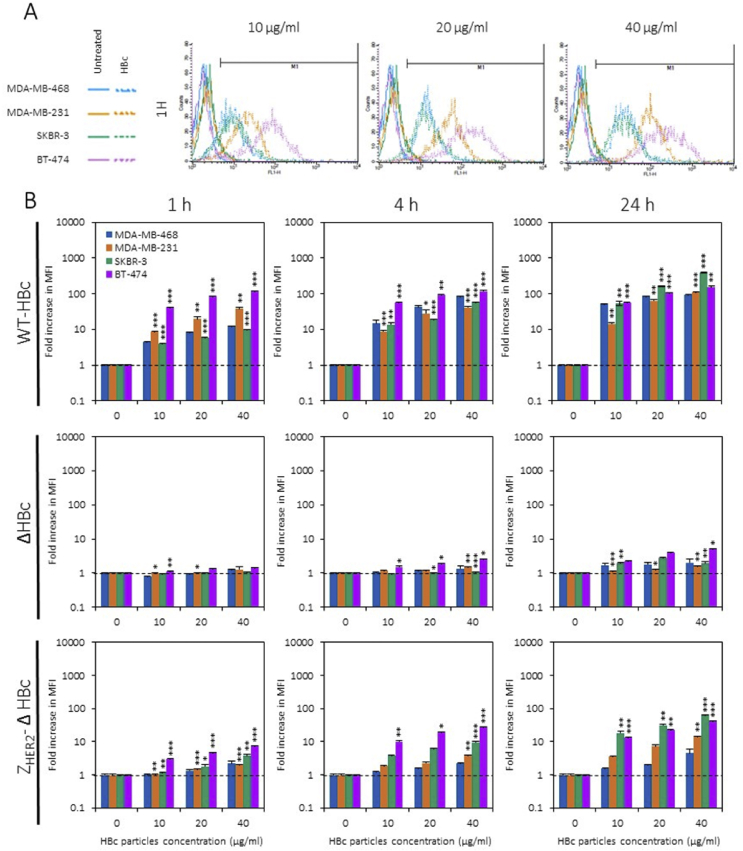
***In vitro* targeting studies using HBc particles. (A)** Fluorescence intensity histogram of cells treated with WT-HBc particles. **(B)** Fold increase in median fluorescence intensity, relative to naïve cells, of cells treated with fluorescently labelled Alexa Fluor™ 488 WT-HBc, ΔHBc or Z_HER2_-ΔHBc particles at increasing concentrations of 10, 20 or 40 μg/mL. Flow cytometry confirmed the non-specific uptake of WT-HBc particles by cancer cell lines in a time- and dose-dependent manner. ΔHBc particles showed a significant reduction in uptake, supporting the hypothesis that the deletion of the arginine-rich domain can reduce the non-specific binding ability of wild type HBc particles. Specific uptake of Z_HER2_-ΔHBc particles in HER2 (+) and (+++) cells is occurred in a time-, dose- and HER2-dependent manner. Values are expressed as fold increase ±SD. *P < 0.05, **P < 0.01, ***P < 0.001, relative to MDA-MB-468 cell line (one-way ANOVA test).

The intracellular uptake of fluorescently labelled Alexa Fluor™ 488 WT-HBc, ΔHBc and Z_HER2_-ΔHBc in breast cancer cells; MDA-MB-468, MDA-MB-231, SKBR-3 and BT-474 was evaluated with flow cytometry using the FL-1 detector. Degree of uptake was expressed as a fold increase in MFI compared to naïve cells. All cells were treated for 1, 4 or 24 h with HBc particles at increasing concentrations of 10, 20 or 40 μg/mL. The fold increase in MFI of cells treated with WT-HBc particles confirmed their non-specific uptake by cancer cells in a time- and dose-dependent manner (Fig. S2, Fig. 4). All cancer cells treated with ΔHBc particles showed very little increase in the fold increase in MFI, indicating a significant reduction in intracellular uptake of the truncated particles, supporting the hypothesis that the deletion of the arginine-rich domain can reduce the non-specific binding ability of WT-HBc particles. In contrast, treatments with Z_HER2_-ΔHBc particles in the HER2-positive cell lines, SKBR-3 (green bars) and BT-474 (purple bars) cells, promoted an apparent fold increase in a time- and dose-dependent manner. In contrast, MDA-MB-231 (orange bars) cells treated with Z_HER2_-ΔHBc particles observed a lower value in fold increase whereas MDA-MB-468 (blue bars) cells exhibited the least (Fig. 4). These results indicate that specific uptake of Z_HER2_-ΔHBc particles in HER2 (+) and (+++) cells occurs in a time- and dose-dependent manner in accordance with HER2 expression levels in cells.

### 3.6 ^99m^Tc radiolabelling of HBc particles and stability studies

**Fig. 5:**
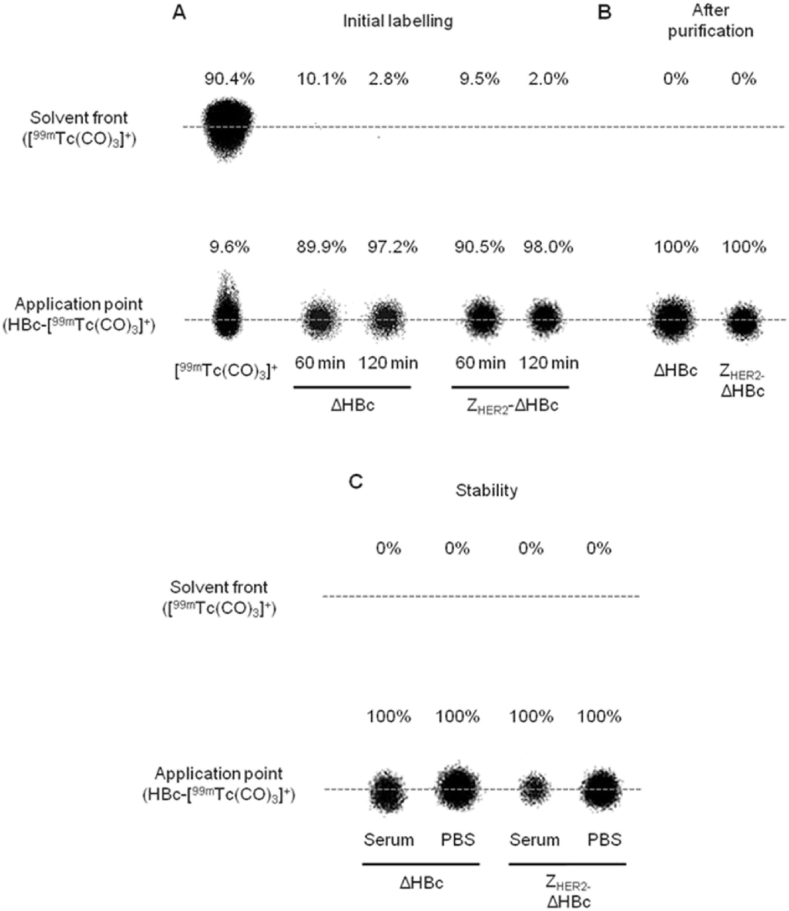
**Radiolabelling efficiency, purification and *in* vitro serum stability of recombinant HBc particles by phosphor imaging. (A)** The radiolabelling reaction was carried out at 37 °C and monitored for 2 h with labelling efficiency determined at 60 and 120 min. 97.2% and 98.0% radiolabelling efficiency was achieved for ΔHBc and Z_HER2_-ΔHBc, respectively. **(B)**^99m^Tc-ΔHBc and ^99m^Tc-Z_HER2_-ΔHBc was purified from free [^99m^Tc(CO)_3_]^+^ and buffer exchanged with PBS buffer using 10 kDa MWCO Vivaspin. Both types of HBc particles exhibited 100% radiolabelling efficiency. **(C)** Serum stability was tested by incubation of ^99m^Tc-ΔHBc and ^99m^Tc-Z_HER2_-ΔHBc in 50% serum or PBS buffer at 37 °C up to 24 h. Both types of HBc particles exhibited 100% stability in serum and PBS indicated by no [^99m^Tc(CO)_3_]^+^ at the solvent front.

^99m^Tc radiolabelling of assembled HBc particles was performed as described by Badar et al. [17] with modifications. [^99m^TcO_4_]^-^ was activated to [^99m^Tc(CO)_3_]^+^ by adding the IsoLink kit followed by heating and adjusting the pH to 7.0–7.5,. NaCl was added upon the mixing of [^99m^Tc(CO)_3_]^+^ with ΔHBc or Z_HER2_-ΔHBc particles to achieve the final [Na^+^] concentration of 0.63 M, to achieve high radiochemical yield (95%) [17]. Radiolabelling efficiency was determined using iTLC. At 60 min post-incubation, both ΔHBc and Z_HER2_-ΔHBc showed radiolabelling efficiency of 89.9% and 90.5%, respectively. The radiolabelling efficiency increased to 97.2% (**p < 0.01) and 98.0% (**p < 0.01) for ΔHBc and Z_HER2_-ΔHBc, respectively, after 120 min post-incubation (Fig. 5A). Radiolabelled ^99m^Tc-ΔHBc and ^99m^Tc-Z_HER2_-ΔHBc were then purified with 10 kDa MWCO Vivaspin to remove any unbound [^99m^Tc(CO)_3_]^+^, and exchange the buffer with PBS to achieve isotonic condition. As shown in Fig. 5B, no unbound [^99m^Tc(CO)_3_]^+^ was observed in both ^99m^Tc-ΔHBc and ^99m^Tc-Z_HER2_-ΔHBc dispersions. A good radiolabelling stability of ^99m^Tc-ΔHBc and ^99m^Tc-Z_HER2_-ΔHBc in PBS and 50% serum incubated at 37 °C for 24 h was also obtained (Fig. 5C).

### 3.7 Whole body SPECT/CT imaging and organ biodistribution of IP-administered HBc particles in the IP tumour model

Two different tumour-bearing mice models, through intraperitoneal (IP) and mammary fat pad (MFP) inoculation were used for *in vivo* studies (See Supplement Information Fig. S3). Solutions containing only [^99m^Tc(CO)_3_]^+^ was used as control (See Supplement Information, Figs. S4-S6). Short blood circulation times, and hence poor tumour accumulation, were observed in mice given radiolabelled ^99m^Tc-ΔHBc or ^99m^Tc-Z_HER2_-ΔHBc *via* intravenous injection in both tumour models (Figs. S7–S8). Local administration into MFP tumours and IP injection for the IP tumour model were therefore attempted. SPECT/CT imaging was performed followed by *ex vivo* gamma counting of major organs. Three-dimensional reconstruction of the whole animal by SPECT/CT imaging was conducted immediately after injection (<30 min), 4 h and 24 h post-injection. Quantitative studies by gamma counting were performed at 24 h post-injection.

**Fig. 6:**
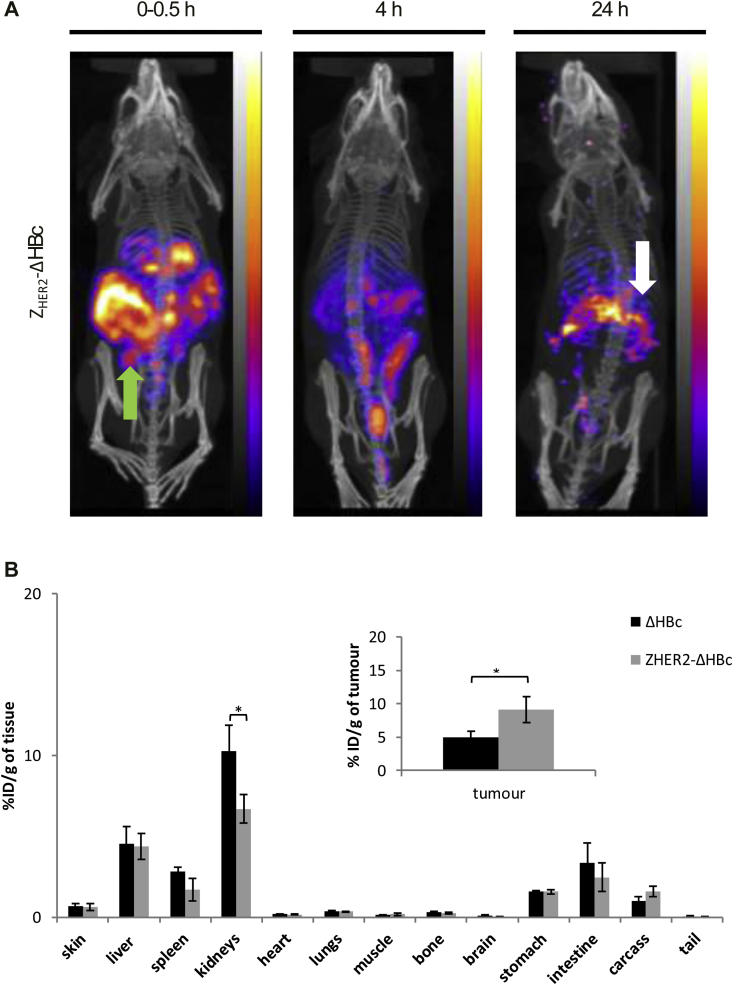
***In vivo* SPECT/CT imaging and organ biodistribution studies of IP-administered**^**99m**^**Tc-HBc particles in MDA-MB-435-MLE IP tumour-bearing NSG mice**. Mice were intraperitoneally injected with ^99m^Tc-ΔHBc (black bars) or ^99m^Tc-Z_HER2_-ΔHBc (grey bars) at a dose of 50 μg protein/mouse (4–6 MBq per mouse). Organs were excised at 24 h post-injection for gamma counting. **(A)** Whole body SPECT/CT imaging at 0–30 min, 4 and 24 h post-injection of ^99m^Tc-Z_HER2_-ΔHBc. Green arrow indicates the location of the administration. White arrow indicates accumulation in the tumour at 24 h post-injection. **(B)** Organ biodistribution profile with values expressed as %ID/g of tissue. Inset shows the %ID/g tumour uptake. A significant increase in tumour uptake was shown in the IP model treated with ^99m^Tc-Z_HER2_-ΔHBc compared with mice treated with ^99m^Tc-ΔHBc (*p < 0.05). Results are expressed as mean ± SD (n = 3). (For interpretation of the references to colour in this figure legend, the reader is referred to the web version of this article.)

Following IP injection, both types of HBc particles exhibited a wide-spread pattern in the abdominal cavity at 0–30 min, with signals started to appear in bladder at 4 h post-injection. Interestingly, stronger signals were associated with the tumour masses in the case of ^99m^Tc-Z_HER2_-ΔHBc compared to ^99m^Tc-ΔHBc = especially at 24 h post-injection (Fig. 6A and Fig. S9A far-right panel, white arrow). Excretion profiles can be seen in Supplementary Information, Fig. S10A. Major organ biodistribution profiles of ^99m^Tc-ΔHBc showed the uptake in kidneys > liver > spleen with 10.27 ± 1.59, 4.54 ± 1.06 and 2.84 ± 0.24%ID/g of tissue at 24 h post-injection, respectively (Fig. 6B, black bars). ^99m^Tc-Z_HER2_-ΔHBc exhibited comparable profile except for the kidney (6.69 ± 0.87%ID/g, *p < 0.05) (Fig. 6B, grey bars). Matching the SPECT/CT results, a significantly higher accumulation in tumour uptake of ^99m^Tc-Z_HER2_-ΔHBc (9.12 ± 0.11%ID/g, *p<0.05) (**grey bars**) was observed compared to ^99m^Tc-ΔHBc (5.00 ± 0.89%ID/g) (**black bars**) at 24 h post-injection (Fig. 6B).

### 3.8 Whole body SPECT/CT imaging and organ biodistribution studies of locally (IT)-administered HBc particles in the MFP tumour model

**Fig. 7:**
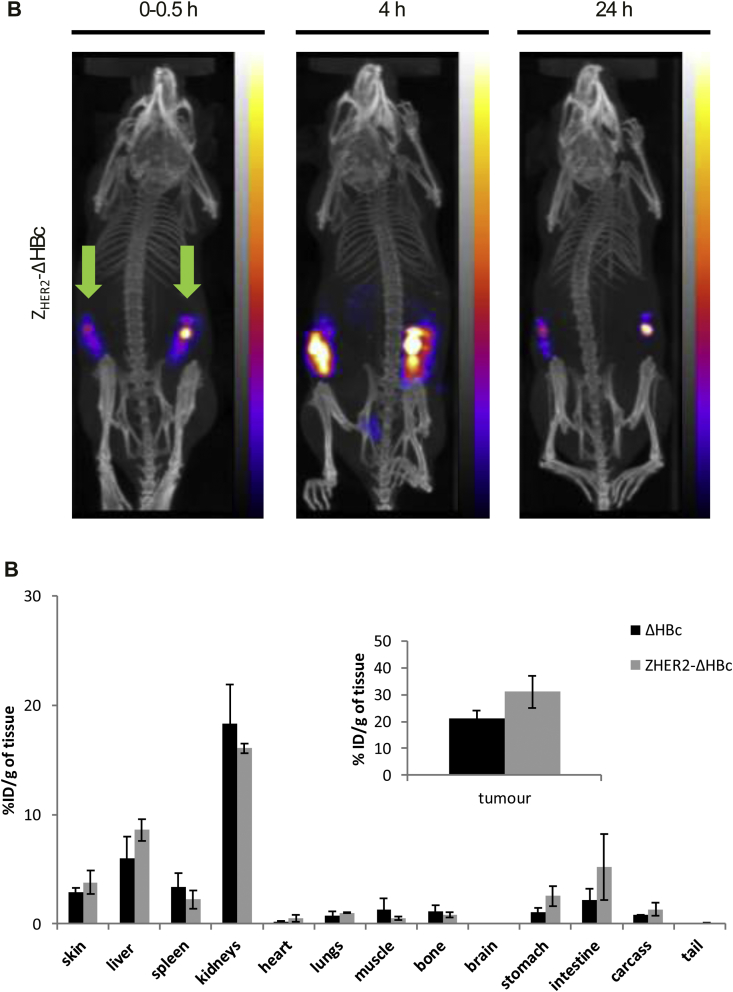
***In vivo* SPECT/CT imaging and organ biodistribution studies of locally-administered**^**99m**^**Tc-HBc particles in MDA-MB-435-MLE MFP tumour-bearing NSG mice**. Mice were intratumourally injected with ^99m^Tc-ΔHBc (black bars) or ^99m^Tc-Z_HER2_-ΔHBc (grey bars) at a dose of 50 μg protein/mouse (4–6 MBq per mouse). Organs were excised at 24 h post-injection for gamma counting. Green arrows indicate the location of the administration. **(A)** Whole body SPECT/CT imaging at 0–30 min, 4 and 24 h post-injection of ^99m^Tc-Z_HER2_-ΔHBc. **(B)** Organ biodistribution profile with values expressed as %ID/g of tissue. Inset shows the %ID/g tumour uptake. No significant increase in tumour uptake was shown in MFP model treated with ^99m^Tc-Z_HER2_-ΔHBc compared with mice treated with ^99m^Tc-ΔHBc (p > 0.05). Results are expressed as mean ± SD (n = 3). (For interpretation of the references to colour in this figure legend, the reader is referred to the web version of this article.)

SPECT/CT imaging exhibited similar biodistribution profiles for ^99m^Tc-ΔHBc (Fig. S9B) and ^99m^Tc-Z_HER2_-ΔHBc (Fig. 7A), where most of the signals appeared in the MFP tumours and some radioactivity was seen in the bladder. Excretion profiles can be seen in Supplementary Information (Fig. S10B). Gamma counting showed accumulation of ^99m^Tc-ΔHBc in the kidneys > liver > spleen with 18.32 ± 3.58, 6.03 ± 1.95 and 3.40 ± 1.28%ID/g of tissue, at 24 h post-injection, respectively (Fig. 7B, black bars). A matching profile was observed for ^99m^Tc-Z_HER2_-ΔHBc (Fig. 7B, grey bars). There was slightly higher but not significant tumour uptake for ^99m^Tc-Z_HER2_-ΔHBc (31.06 ± 6.05%ID/g of tumour) compared to ^99m^Tc-ΔHBc (21.09 ± 3.12%ID/g of tumour) (Fig. 7B, inset).

The overall conclusion from these biodistribution studies is that the IP tumour model seems to be a more suitable model for future *in vivo* cancer therapy studies using HBc particles. Most importantly, the results provided solid evidence on the substantial tumour accumulation and targeted uptake of Z_HER2_-ΔHBc particles in HER2 (+++) tumours *in vivo* over the 24 h period studied.

## 4 Discussion

Virus-like particles (VLPs) have been used as nano-carriers to display foreign epitopes and also deliver small molecules to cells, tissues or organs [24]. The strategy of using these VLPs as drug delivery systems has attracted many researchers' attention to combat many diseases, including cancer [24]. Among the VLPs, HBc particles were one of the first models of ‘displaying nano-carriers’, carrying different desired foreign epitopes on their surface including for vaccination [24], [25]. Much attention has been given to HBc particles due to their high-level production in well-known homologous and heterologous expressions systems, such as *Escherichia coli*, and their correct self-assembly ability into core particles in the absence of any viral components [26], [27], [28], [29]. However, as mentioned earlier, the native or wild type HBc particles are known for their non-specific binding properties, due to the positively-charged arginine-rich domain located at 150–183 aa [7], [10]. To address this issue, Nishimura et al. has developed recombinant HBc particles, where the non-specific binding of wild type HBc particles was reduced by genetically deleting the arginine-rich domain of 150–183 aa region [22], [30]. It has been reported that the arginine-rich domain is not required for correct assembly of HBc particles. Therefore, deletion of this poly-arginine region does not affect their structure and particle assembly [30], [31].

A HER2 targeting system was developed by expressing Z_HER2_ affibodies on the spike of the truncated HBc particles (ΔHBc). The Z_HER2_ affibody was introduced into the major immunodominant region (MIR) of 78–81 amino acids (aa) position of the HBc core protein. This 78-81 aa of MIR region forms the tips on the particles' spikes thus are exposed and accessible on the surface of the HBc particles, making it available for cell-receptor-ligand interaction [32]. In the previous study by Nishimura et al., ΔHBc particles were employed to express HER2 targeting moiety since they lack the arginine rich-domain, which is responsible for the non-specific binding of HBc to a variety of cells. Results in this study showed that non-specific uptake was observed when all four different breast cancer cell lines, MDA-MB-468, MDA-MB-231, SKBR-3 and BT-474 were treated with WT-HBc particles. The arginine-rich domain of HBc particles has been well studied, specifically on its function and effects on the particles' assembly and nucleic acid binding [7], [33], [34]. It has been demonstrated that the arginine-rich domain binds the cell surface heparan sulphate proteoglycan *via* electrostatic interactions. Studies have shown that any protein with either an arginine-rich domain or a protein transduction domain (PTD) will bind the heparan sulphate proteoglycan on a cell surface, resulting in a non-specific binding of native or wild type HBc particles to any cells including cancer cells [35], [36], [37]. It has also been reported that the engineered ΔHBc particles lacking the arginine-rich domain, specifically arginine residues in aa 150–159, could associate and form the spherical particle's structure, although this would reduce its function of cell binding and uptake [22], [38]. This agrees with results obtained in this study where the removal of the entire arginine-rich domain from the HBc particles structure managed to significantly reduce the cell uptake of HBc particles when the cancer cells were treated with ΔHBc particles.

The main concern of using HBc particles or any virus-like particles is the immunogenicity. The MIR region is known for its function to elicit a strong antibody response including a strong B cell, T helper cell and cytotoxic T cell response. An introduction of targeting moieties at the MIR region will not only optimally remove a strong antibody response against the native MIR; but it also allows the inserted sequences to be available at the surface of the particle to bind specifically its target receptor [39], [40].

Our work is different from previously reported study [22], in that, an optimised expression protocol was used for the expression, purification and assembly of HBc particles. The results showed that treating MDA-MB-468, MDA-MB-231, SKBR-3 and BT-474 cells at increasing concentrations of HBc particles at 10, 20 or 40 μg/mL resulted in specific uptake in a time- and dose-dependent manner. This is an extension of Nishimura et al.*’s* work in which HBc particles at concentrations up to 10 μg/mL were tested [22].

A variety of nanoparticles including VLPs are being developed for biomedical applications [41], [42], [43], [44], [45], [46]. However, information regarding their biological behaviours *in vivo* is still limited. Interventions, such as routes of administration, biodistribution patterns and pharmacological profiles, are important for the design and development of successful bio-nanoparticles for future clinical and cancer therapeutic studies [47], [48]. In the current study, we analysed the behaviour of recombinant HBc particles, specifically truncated recombinant HBc particles (ΔHBc) and HER2-targeting Z_HER2_ affibodies-expressing recombinant HBc particles (Z_HER2_-ΔHBc), in two HER2 (+++) MDA-MB-435-MLE tumour bearing NSG mice models, namely, IP and MFP tumour models. While orthotopic or ectotopic implantation of cancer cells in the MFP provides a well-defined model to mimic early stages of breast cancer growth [49], [50], IP tumour mouse model offers close representation of clinical disease progression of tumour, including metastatic dissemination [51], [52]. HBc particles were administered in these two models *via* the systemic (intravenous or intraperitoneal injection) or local (intratumoural) routes.

In order to investigate the biodistribution qualitatively and quantitatively, HBc particles were radiolabelled with the radionuclide technetium-99 m (^99m^Tc). ^99m^Tc is used to trace small protein (<55 kDa) with a half-life sufficiently long 6 h) to allow for radiolabelling and biodistribution studies to be carried out. It also has favourable photon energy for imaging while minimising patient radiation-absorbed dose. Radiolabelling can be achieved using site-specific labelling methods that exert maximum control over the number and site modification(s) to the molecule, without altering the protein function [53]. In this study, the His-tag located at the C terminal of the recombinant HBc particles was used to site-specifically radiolabel the protein with 99mTc-tricarbonyl ([^99m^Tc(CO)_3_]^+^). This method was firstly developed by Waibel et al. [54] then further modified by Badar et al. [53]. The His-tag was originally used to facilitate purification of the HBc particles using metal chelate-based affinity chromatography [55]. Histidine has been a favourable binding ligand to [^99m^Tc(CO)_3_]^+^ compared to other amino acids as the labelling efficiency and stability increase with the increased number of histidines present [56]. Using the technique reported by Badar et al., radiolabeling efficiency of >95% was achieved for both particles. High radiolabelling efficiency is ideal (∼95%), as low labelling efficiency can hamper quantitative measurements and imaging sensitivity. Furthermore, an additional purification step prior to imaging will be required which may cause excessive dilution and losses of the protein due to its irreversible binding to columns and filters [53].

The clearance kinetics of proteins and VLPs have been reported in several studies [3], [57], [58], [59]. In general, particulate materials including viral proteins are removed from the blood circulation relatively quickly by the reticulo-endothelial system (RES) (e.g. liver and spleen) [60], [61]. For example, Singh et al. reported that the retention time of lanthanide-*Cowpie mosaic virus* (CPMV) particles in bloodstream was about 15–30 min [3]. *Adenovirus* (Ad) particles, reported by Green et al. also appear to be sequestered from circulation in part by RES in mice, presumably by interaction with the scavenger receptors or platelets [62]. Here, we also observed the relatively rapid clearance from blood stream of intravenously-administered HBc particles in both intraperitoneal and mammary fat pad tumour models, similar to CPMV and Ad particles. The lack of significant differences in tumours uptakes observed between the two types of HBc particles in both tumour models might be due to their short blood circulation times. This rapid clearance of HBc particles suggests that modification with polyethylene glycol (PEG) or other masking agents might be required to prolong the blood circulation times of the particles [63], [64]. Raja et al. reported that CPMV particles coated with PEG managed to reduce the particle's uptake in liver and spleen, causing a prolonged blood circulation time in mice [65].

Quantitative studies by gamma counting exhibited significant tumour accumulation by IP or IT injection of HBc particles. A significant tumour targeting effect was observed in the case of IP injection of ^99m^Tc-Z_HER2_-ΔHBc particles in the IP tumour model. The peritoneal cavity is the site of disease in several cancers, including ovarian, gastrointestinal and peritoneal mesothelioma. After injection into the peritoneal cavity, a drug or a nanocarrier, is taken up by the surrounding peritoneal tissues, and from there spread into other body compartments *via* the circulation [66]. Using the IP route not only avoids systemic distribution of the drug, but also provides high concentrations, locally, which are then able to act on the peritoneal tissues directly [67]. Many studies have shown the use of the IP route for cancer therapy including chemotherapy. Dedrick et al. demonstrated the pharmacokinetic rationale for peritoneal drug administration in the treatment of ovarian cancer, as the tumour can be exposed to higher concentrations of chemotherapeutics for prolonged periods of time if given intraperitoneally, rather than intravenously [68]. In addition to administering drugs, the interest of using the peritoneal cavity to deliver gene therapy agents has been increasing. Conclusive results have been achieved in ovarian and pancreatic tumour bearing mice models with adenoviral and retroviral particles, respectively [69], [70]. Another study using a mice model of ovarian cancer looked at the effects of small interfering RNA (siRNA), incorporated into liposomal carriers. The results showed a combination of IP administration with paclitaxel drugs was able to reduced tumour growth compared to paclitaxel/control siRNA by 48–81%, which was comparable to mice treated with paclitaxel and siRNA through IV administration route [71]. These findings suggest the therapeutic potential of IP route for drug delivery, which may have an important role in the treatment of intra-abdominal malignancies in the future.

However, we also observed a good retention of the non-targeted HBc particles in the IP tumour mice model even at 24 h post treatment. The chosen *in vivo* tumour line, MDA-MB-435-MLE, exhibited a vascularised tumour property. This might be one of the factors that contributed to the retention of the ΔHBc particles. However, the HER2-targeted HBc particles (Z_HER2_-ΔHBc) exhibited a significantly higher retention in the tumour (*p < 0.05) compared to the ΔHBc particles, so that we could conclude that our Z_HER2_-ΔHBc particles exhibited a significant enhancement in uptake in HER2-over-expressing tumours.

In IT-administered HBc particles in mammary fat pad tumour model, we observed good retention of both HBc particles but also [^99m^Tc(CO)_3_]^+^ in the tumours. It has been discussed about the challenges encountered in intratumoural administration of drug delivery agents [72], [73]. Despite the good retention in the tumour, this may not necessarily translate to good uptake into cancer cells. Barriers to homogenous distribution of nanoparticle in the tumour tissues include the extracellular matrix [74], [75], relatively high interstitial fluid pressure [76] and affinity of the particles for the tumour cells in the peripheral region of a solid tumour [77]. Our study suggests that IP administration of the engineered HBc particles results in high accumulation of intraperitoneal tumour models.

On the other hand, many articles have referred to and used MDA-MB-435 (parental cell line of MDA-MB-435-MLE) as a breast cancer tumour model. However, recent studies by Prasad et al. showed that the MDA-MB-435 cell line was not a breast cancer cell but instead a type of melanoma cell line [78]. Therefore, this cell line will not be affected by the difference level of hormones.

## 5 Conclusions

In summary, we developed genetically modified HBc particles that specifically recognise and target human epidermal growth factor receptor-related (HER2)-expressing cancer cells. *In vitro* studies showed specific uptake of Z_HER2_-ΔHBc particles in HER2 expressing cancer cells. It is envisaged that this HER2-targeting HBc can be loaded with drugs or any small molecules such as siRNA *via* the dis-assembly/re-assembly processes. *In vivo* studies showed that the combination of IP administration and the use of targeted HBc particles (^99m^Tc-Z_HER2_-ΔHBc) resulted in significantly enhanced tumour accumulation in the intraperitoneal tumour model, suggesting selective uptake of Z_HER2_-ΔHBc particles in HER2 (+++) tumours *in vivo.* Findings from this work offer fundamental knowledge on the biodistribution of newly reported recombinant HBc particles designed for local delivery of nucleic acids to intraperitoneal cancer.

## Figures and Tables

**Table 1:** Yield and size characterisation of WT-HBc, ΔHBc and Z_HER2_-ΔHBc particles.

HBc particles	Protein yield (mg)^a^^,^^c^	OD_260_:OD_280_^a^^,^^c^	Size ± SD (nm)^b^^,^^d^
WT-HBc	3.21 ± 0.21	0.58 ± 0.01	33.77 ± 4.58
ΔHBc	3.73 ± 0.58	0.62 ± 0.06	30.24 ± 2.87
Z_HER2_- ΔHBc	3.13 ± 0.23	0.56 ± 0.05	32.41 ± 2.33

a Values were obtained with Nanodrop from 1 L bacteria containing media.

b Values were obtained with Atomic Force Microscopy (AFM).

c Results are expressed as an average ± SD (n = 3).

d Results are expressed as an average ± SD (n = 50).
