# Supplementary material for: Engineering hepatitis B virus core particles for targeting HER2 receptors in vitro and in vivo
Source: Biomaterials. 2017 Mar;120:126–38. doi: 10.1016/j.biomaterials.2016.12.012 (PMC5300899; doi:10.1016/j.biomaterials.2016.12.012)
Supplement: Supplementary file 1 [file mmc1.pdf]

Supporting Information

Engineering Hepatitis B Virus Core Particles for Targeting HER2 Receptors *In Vitro* and *In Vivo*

Izzat Fahimuddin Bin Mohamed Suffian[1], Julie Tzu-Wen Wang[1], Naomi O. Hodgins[1], Rebecca Klippstein Martin[1], Mitla Garcia-Maya[2], Paul Brown[2], Yuya Nishimura[3], Hamed Heidari[4], Sara Bals[4], Jane Sosabowski[5], Chiaki Ogino[3], Akihiko Kondo[3], Khuloud T. Al-Jamal[1]*

[1] *Institute of Pharmaceutical Science, King's College London, Franklin-Wilkins Building, 150 Stamford Street, London SE1 9NH, UK.*

[2] *Randall Division of Cell & Molecular Biophysics, King's College London, New Hunt's House, London SE1 1UL, UK.*

[3] *Department of Chemical Science and Engineering, Graduate School of Engineering, Kobe University, 1-1 Rokkodai, Nada, Kobe 657-8501, Japan.*

[4] *Electron Microscopy for Materials Science (EMAT), University of Antwerp, Groenenborgerlaan 171, B-2020, Antwerp, Belgium.*

[5] *Centre for Molecular Oncology, Bart's Cancer Institute, Queen Mary University of London, London, EC1M 6BQ, UK*

* Corresponding author

Prof. Khuloud T. Al-Jamal

Institute of Pharmaceutical Science, King's College London, Franklin-Wilkins Building,

150 Stamford Street, London SE1 9NH, UK.

Tel: +44(0)20-7848-4525

1. Supporting Materials

Ampicillin sodium, AIM-terrific broth base including trace elements, Tris base Ultra-Pure, EDTA disodium and DTT dithiothreitol were obtained from ForMedium™ (UK). Calcium chloride (anhydrous) and urea (carbamide) were obtained from Melford (UK). Imidazole, Glycerol, 2-mercapoethanol, *N*, *N*, *N'*, *N'*-tetramethylethylenediamine, sodium chloride, agarose, ribonuclease A (from bovine pancreas) and Bromophenol Blue sodium salt were obtained from Sigma Life Science (UK). cOmplete™ ULTRA Tablets, glass vials Protease Inhibitor Cocktail and cOmplete™ His-Tag Purification Resin were from Roche (Germany). Sodium hydrochloride and methanol were obtained from Fisher Scientific (UK). Acetic acid ≥99.0% (T) and skimmed milk powder were obtained from Fluka Analytical (Switzerland). Triton® X-100, sodium carbonate, ammonium persulfate and Bovine Serum Albumin lyophilized powder, ≥96% (agarose gel electrophoresis) were obtained from Sigma-Aldrich (Germany). Citric acid monohydrate and Brilliant Blue R were obtained from Sigma (UK). Sodium dodecyl sulphate was obtained from BDH Laboratory (UK). Acrylamide/Bis Solution (30%), 37.5:1, Precision Plus Protein™ Dual Xtra Standards, Precision Protein StrepTactin-HRP Conjugate and Clarity Western ECL Substrate were obtained from Bio-Rad Laboratories (USA). Mouse Anti-Hepatitis B Virus Antibody, core antigen (ayw), clone 10E11 was from Merck Millipore (USA). Hydrochloric acid, for analysis, fuming, 37% solution in water was obtained from Aeros Organic (Germany). UltraPure™ Phenol:Chloroform:Isoamyl Alcohol was obtained from Invitrogen (USA). Anti 6-His affinity purified was obtained from Bethyl Laboratories Inc. (USA). Newborn Calf Serum, Heat Inactivated was obtained from First Link Limited (UK). PE Mouse Anti-Human HER-2/neu and PE Mouse IgG1, κ Isotype Control were from BD Biosciences (USA). Alexa Fluor® 488 NHS Ester (Succinimidyl Ester), MEM GlutaMAX™ Supplement, DMEM high glucose pyruvate, Advanced RPMI 1640 Medium, PBS (10X) pH 7.4, Trypsin-EDTA (0.05%) phenol red, Penicillin-Streptomycin (10,000 U/mL), L-Glutamine (200 mM), GlutaMAX™ Supplement,

SnakeSkin™ Dialysis Tubing 10K MWCO 35 mm and Pierce™ 16% Formaldehyde (w/v) Methanol-free were from Thermo-Fisher Scientific (USA). IsoLink kit was obtained from Mallinckrodt Medical BV (Netherlands). $[^{99m}TcCO_4]^-$ solution was obtained from Mallinckrodt Pharmaceuticals (Netherlands). XenoLight™ D-luciferin potassium salt was obtained from Perkin Elmer (EU). *Escherichia Coli*, BL21 (DE3) [F^- ompT hsdSB (rB^- mB^-) gal dcm (DE3)] was obtained from Dr. Mitla Garcia Maya, Randall Division of Cell and Molecular Biophysics, King's College London, UK. Plasmids pET-22b(+)-dc183_WT-His6, pET-22b(+) -dc149-His6 and pET-22b(+)-dc149-Z342-His6 were obtained from Associate Professor Chiaki Ogino, Chemical and Science Engineering Department, Kobe University, Japan.

2. Supporting Methods

2.1 Cell culture

A panel of cell lines, of varied degree of HER2 expression, was used in this study. The BT-20 human breast carcinoma (BT-20; ATCC, HTB-19) was cultured in MEM media supplemented with 15% fetal bovine serum (FBS), 50 U/ml penicillin, 50 µg/ml streptomycin, 1% L-glutamine, at 37 °C in 5% CO_2. The IGROV-1 human ovarian adenocarcinoma (IGROV-1; RRID, CVCL_1304) and SKOV-3 human ovary adenocarcinoma (SKOV-3; ATCC, HTB-77) were cultured in DMEM media supplemented with 10% FBS, 50 U/ml penicillin, 50 µg/ml streptomycin, 1% L-glutamine, at 37 °C in 5% CO_2. The HeLa human cervix adenocarcinoma (HeLa; ATCC, CCL-2) were cultured in DMEM media supplemented with 10% FBS, 50 U/ml penicillin, 50 µg/ml streptomycin, 1% L-glutamine, at 37 °C in 5% CO_2. All cells were routinely grown in 75 cm^2 canted-neck tissue culture flasks and passaged twice a week using Trypsin/EDTA at 80% confluency.

2.2 Determination of HER2 receptors expression levels in cancer cell lines

Cells were cultured in complete media in 25 cm^2 canted-neck tissue culture flasks. When cells reached 80% confluency, cells were washed twice with PBS buffer; trypsinised and 1 x 10^6 cells were transferred to assay tubes for the subsequent immunostaining. Cells were fixed in 4 % PFA for 10 min at room temperature, washed in PBS buffer and incubated with 150 µl of blocking buffer (1% BSA in PBS buffer) for 30 min at room temperature. After this period of time, cells were washed with PBS buffer and incubated with 20 µl of PE Mouse Anti-Human HER-2neu antibody (BD Biosciences, USA) or 20 µl of Isotype Mouse BALB/c IgG_1, κ for 30 min in dark at room temperature. Finally, cells were washed with PBS buffer. PE fluorescence was analysed by flow cytometry using a BD FACS Calibur™ flow cytometer (BD Biosciences, USA). A total of 10,000 cells were gated and fluorescence was analysed in triplicates for each condition using the FL-2 detector and BD CellQuest software (BD Biosciences, USA).

3. Supporting Results

3.1 Characterisation of cancer cells for HER2 expression

HER2 expression levels in 9 cancer cell lines, including MDA-MB-468, MDA-MB-231, IGROV-1, HeLa, BT-20, SKOV-3, MDA-MB-435-MLE, SKBR-3 and BT-474 cells, were assessed using flow cytometry. Expression levels were assessed by measuring the median fluorescent intensity (MFI) using FL-2 detector. Cell lines were stained with PE mouse anti-human HER2/neu primary antibody. κ isotype was used as a negative control.

As shown in **Figure S1A**, MDA-MB-468 cells showed no difference in the mean fluorescence intensity (MFI) between cells stained with the mouse anti-human HER2/neu primary antibody and naïve (grey colour) or isotype control cells (green line); suggesting that

MDA-MB-468 cells are HER2 negative. On the contrary, MDA-MB-231, IGROV-1, HeLa, BT-20, SKOV-3, MDA-MB-435-MLE, SKBR-3 and BT-474 were confirmed HER2 positive, as shown by the increase in MFI of cells, stained with mouse anti-human HER2/neu (FL2) (purple line) (**Figure S1A**). Based on MFI values, HER2 expression levels on cells were ranked in the following order: MDA-MB-468 < MDA-MB-231 < IGROV-1 < HeLa < BT-20 < SKOV-3 < MDA-MB-435-MLE < SKBR-3 < BT-474. Cells were classified as negative (-) (MDA-MB-468), lowly expressed (+) (MDA-MB-231, IGROV-1, HeLa, BT-20) or overly expressed (+++) (SKOV-3, MDA-MB-435-MLE, SKBR-3, BT-474) for HER2 receptors (**Figure S1B**). Only MDA-MB-468, MDA-MB-231, SKBR-3 and BT-474 cell lines were chosen as representative cell lines for further cellular uptake studies.

3.2 Fluorescence labelling of HBc particles

All HBc particles were fluorescently labelled with Alexa Fluor™ 488 dye. Fluorescence was measured with excitation at 485 nm and emission at 520 nm, using BMG FLUOstar Omega fluorometer. As shown in **Figure S2**, HBc particles showed higher fluorescence intensity than ΔHBc and Z_{HER2}-ΔHBc particles (**Figure S2**).

3.3 *In vivo* monitoring of highly-expressing HER2 (HER2 (+++)) xenograft tumour models by bioluminescence imaging

In order to study the biodistribution of HBc particles in an *in vivo* model, two tumour mice models were prepared; intraperitoneal and mammary fat pad tumour models. NSG mice were inoculated with luciferase-expressing MDA-MB-435-MLE cells, either intraperitoneally or subcutaneously into the mammary fat pad, to generate intraperitoneal or mammary fat pad tumour models, respectively. Tumour growth in each model was monitored by bioluminescence *in vivo* imaging, every 7 days after inoculation. Ventral images of a representative NSG mouse from both tumour models are shown in **Figure S3**. The

intraperitoneal model showed tumour growths at multiple sites around the abdominal cavity, including the spleen, intestines and male reproductive organs (**Figure S3A**). Meanwhile, localised bioluminescent signals were observed at mammary fat pads on both sides in the mammary fat pad model (**Figure S3B**).

3.4 Blood clearance, excretion profiles and organ biodistribution profile of systemically-administered [$^{99m}Tc(CO)_3]^+$ particles by gamma counting

Blood sampling at early time points demonstrated rapid clearance from the blood with values of ~50% and ~30%ID in blood detected at 30 min-post injection, in intraperitoneal and mammary fat pad tumour models, respectively (**Figure S4A**). At 24 h, these values dropped to ~30% and ~10%ID in blood for intraperitoneal and mammary fat pad tumour models, respectively (**Figure S4A**). High radioactivity eliminated into urine was observed for [$^{99m}Tc(CO)_3]^+$ in both intraperitoneal and mammary fat pad tumour models with ~18-28%ID eliminated/mouse (**Figure S4B**). A similar excretion profile with HBc particles in faeces was observed for [$^{99m}Tc(CO)_3]^+$ in both models.

[$^{99m}Tc(CO)_3]^+$ was observed to accumulate in multiple organs, with the kidneys (8.45 ± 3.53%ID/g) and liver (8.06 ± 1.71%ID/g) exhibiting the highest accumulation followed by the intestines (4.69 ± 0.29%ID/g), stomach (4.19 ± 1.14%ID/g), lungs (3.40 ± 1.15%ID/g), spleen (2.97 ± 0.28%ID/g) and heart (1.76 ± 0.54%ID/g) (**Figure S4C, black bars**). Moreover, tumour accumulation was found to be at 1.44 ± 0.17%ID/g of tumour (**Figure S4C, black bar**).

[$^{99m}Tc(CO)_3]^+$ showed a similar accumulation profile in systemically-administered mammary fat pad tumour model (**Figure S8C, grey bars)** to that obtained in intraperitoneal model. Tumour uptake was 0.82 ± 0.20%ID/g of tumour (**Figure S8C, grey bars**).

3.5 *In vivo* whole body SPECT/CT imaging, excretion profiles and organ biodistribution profile of locally-administered $[^{99m}Tc(CO)_3]^+$ particles by gamma counting

In intraperitoneal tumour-bearing mice, whole body SPECT/CT imaging of $[^{99m}Tc(CO)_3]^+$ exhibited a different profile to the HBc particles, in which, an intense radioactivity signals were observed in heart, lungs, liver and bladder. $[^{99m}Tc(CO)_3]^+$ signals were mostly cleared at 24 h post-injection (**Figure S5A)**. Meanwhile, the excretion profile exhibited low radioactivity eliminated in urine (3.17 ± 0.14%ID eliminated/mouse) and faeces (0.65 ± 0.83%ID eliminated/mouse) for $[^{99m}Tc(CO)_3]^+$ (**Figure S5B**). Very low organ accumulation was observed with the uptake of $[^{99m}Tc(CO)_3]^+$ in the intestines (3.55 ± 1.89%ID/g), followed by the liver (2.49 ± 0.39%ID/g), kidneys (2.38 ± 0.13%ID/g), stomach (2.29 ± 1.02%ID/g) and spleen (1.14 ± 0.46%ID/g) (**Figure S5C**). A relatively low tumour accumulation of $[^{99m}Tc(CO)_3]$ (2.32 ± 0.84%ID/g) was observed (**Figure S5C**).

In the whole body SPECT/CT imaging of mammary fat pad tumour-bearing mice, $[^{99m}Tc(CO)_3]^+$ exhibited a similar biodistribution profile to ^{99m}Tc-ΔHBc and ^{99m}Tc-Z_{HER2}-ΔHBc, where by most of the signals were retained in the tumour (**Figure S6A**). No significant radioactivity signals were observed in any organs except bladder, which indicated that some of the particles reached the systemic circulation prior to elimination in the urine. As shown in **Figure S6B,** $[^{99m}Tc(CO)_3]^+$ also resulted in a similar excretion profile to HBc particles**,** with 33.38 ± 5.66% and 1.36 ± 0.11%ID eliminated/mouse in the urine and faeces, respectively. Organ accumulation of $[^{99m}Tc(CO)_3]^+$ was highest in kidneys, followed by liver and spleen with 14.49 ± 3.55, 11.46 ± 3.28 and 3.49 ± 0.37%ID/g of tissue at 24 h post-injection, respectively (**Figure S6C**). $[^{99m}Tc(CO)_3]^+$ exhibited high tumour uptake of ~34.77 ± 15.70%ID/g (**Figure S6C**).

3.6 Blood clearance, excretion profiles and organ biodistribution profile of systemically-administered HBc particles by gamma counting

Quantitative studies by gamma counting were performed to assess the pharmacokinetic profile and organ biodistribution of HBc particles 24 h post-injection. Radiolabelled ^{99m}Tc-ΔHBc and ^{99m}Tc-Z_{HER2}-ΔHBc particles were injected *via* the tail vein (systemic route) in both of the tumour models. Blood sampling at early time points demonstrated fast clearance from the blood with values of ~7.0-7.5% and ~4.0-5.5%ID in blood detected in blood at 30 min-post injection, in the intraperitoneal (**Figure S7A**) and mammary fat pad (**Figure S7B**) tumour models, respectively. At 24 h, these values dropped to ~4.0-5.0% and ~0.5-1.5%ID in blood for the intraperitoneal (**Figure S7A**) and mammary fat pad (**Figure S7B**) tumour models, respectively. No significant difference in the blood clearance profiles between the ^{99m}Tc-ΔHBc and ^{99m}Tc-Z_{HER2}-ΔHBc particles was observed. Relatively low levels of radioactivity suggest that both HBc particles have a short blood circulation time in mice. Animals were also housed in metabolic cages for urine and faeces collection for 24 h to assess excretion profile. Approximately ~10-20% and ~1.0-2.0%ID was eliminated in urine and faeces within 24 h for both particles ($p > 0.05$) (**Figure S7C, D**).

In the intraperitoneal tumour mouse model, accumulation of ^{99m}Tc-ΔHBc was observed in kidneys > liver > spleen followed by lungs at %ID/g values of 13.90 ± 1.08, 9.88 ± 1.24, 9.27 ± 0.57 and 3.41 ± 0.40% at 24 h post-injection (**Figure S8A, black bars**). A similar trend in organ accumulation profile was observed for ^{99m}Tc-Z_{HER2}-ΔHBc (**Figure S8A, grey bars).** The mammary fat pad tumour mouse model exhibited comparable organ biodistribution profiles to those observed in intraperitoneal tumour mouse model (**Figure S8B**). A

statitiscally significant higher lung (*$p < 0.05$) and lower kidney (**$p < 0.01$) uptakes were observed for ^{99m}Tc-Z_{HER2}-ΔHBc compared to ^{99m}Tc-ΔHBc.

Tumour uptake of systemically-administered HBc particles exhibited low uptake with ~ 0.5% and ~0.2% achieved in the intraperitoneal and mammary fat pad tumour model, respectively, with no significant differences observed between ^{99m}Tc-ΔHBc and ^{99m}Tc-Z_{HER2}-ΔHBc (**Figure S8**).

3.7 Excretion profiles of locally-administered HBc particles by gamma counting

In the intraperitoneal tumour-bearing mice model, approximately ~4-10% and ~0.2-2.0 %ID was eliminated into urine and faeces after 24 h for both particles with no significant differences between the type of particles ($p > 0.05$) (**Figure S9A**). In contrast, in the mammary fat pad tumour-bearing mice model, higher radioactivity was observed in urine for both HBc particles after 24 h (~17-22%) than found with the intraperitoneal model. Approximately ~20-30% and ~1.6-1.7 %ID was eliminated into urine and faeces after 24 h for both particles ($p > 0.05$) (**Figure S9B**).

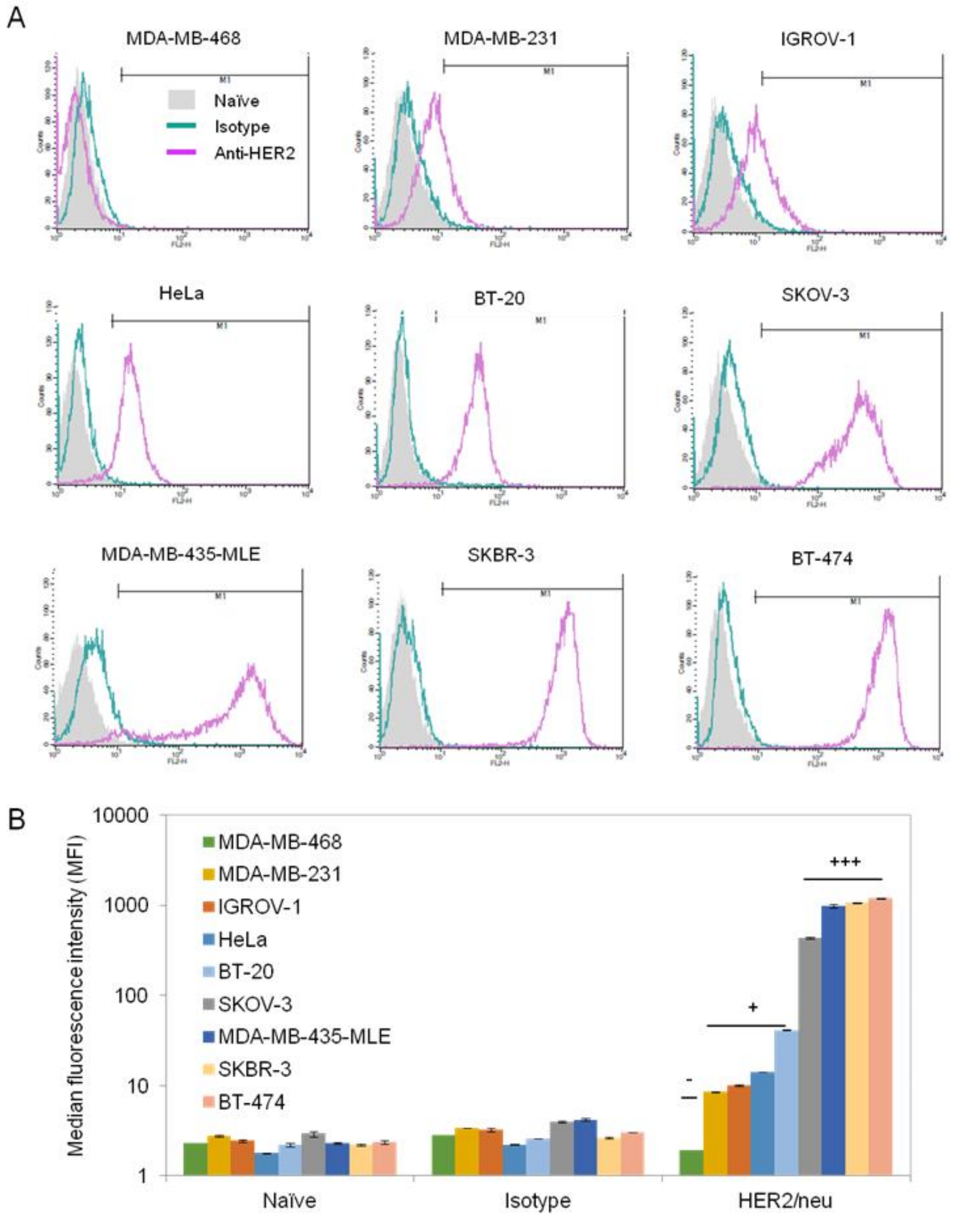

Figure S1. HER2 expression levels in cancer cell lines by flow cytometry. (A) Fluorescence intensity histograms (FL2) and **(B)** Median fluorescence intensity (MFI) analyses. Cell lines were stained with PE mouse anti-human HER2/neu primary antibody for assessing the expression level of HER2 receptors on cell membrane surface. PE mouse anti-human IgG1, κ isotype was used to account for non-specific binding to the IgG. HER2

expression levels on cells are in the following order: MDA-MB-468 > MDA-MB-231 > IGROV-1 > HeLa > BT-20 > SKOV-3 > MDA-MB-435-MLE > SKBR-3 > BT-474. Cells were classified as negative (-), low expression (+) or over-expression (+++) for HER2 receptors.

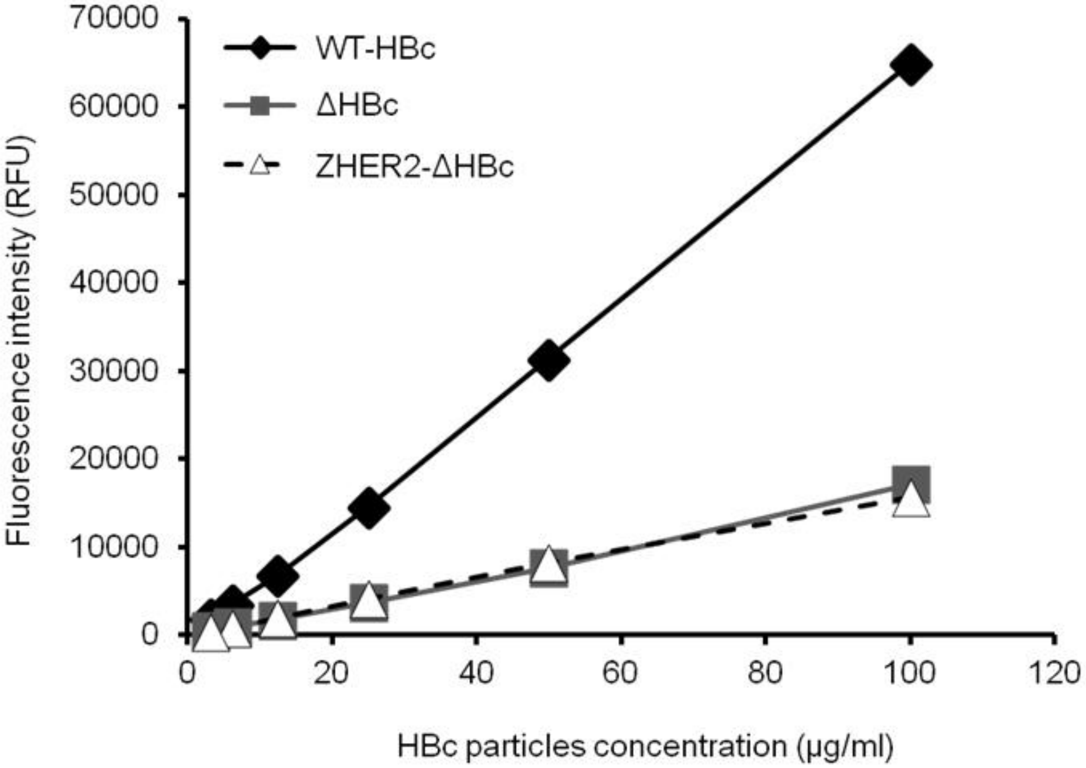

Figure S2. Standard curve of Alexa Fluor™ 488-labelled HBc particles by fluorometry. WT-HBc, ΔHBc and Z_{HER2}-ΔHBc particles were fluorescently labelled with Alexa Fluor™ 488 dye and measured for fluorescence at 485nm and 520 nm excitation and emission wavelengths, respectively. Measurements were performed at 25˚C using a BMG FLUOstar Omega fluorometer. Error bars are too small to be seen. Higher labelling efficiency was obtained for WT-HBc than ΔHBc and ZHER2-ΔHBc particles.

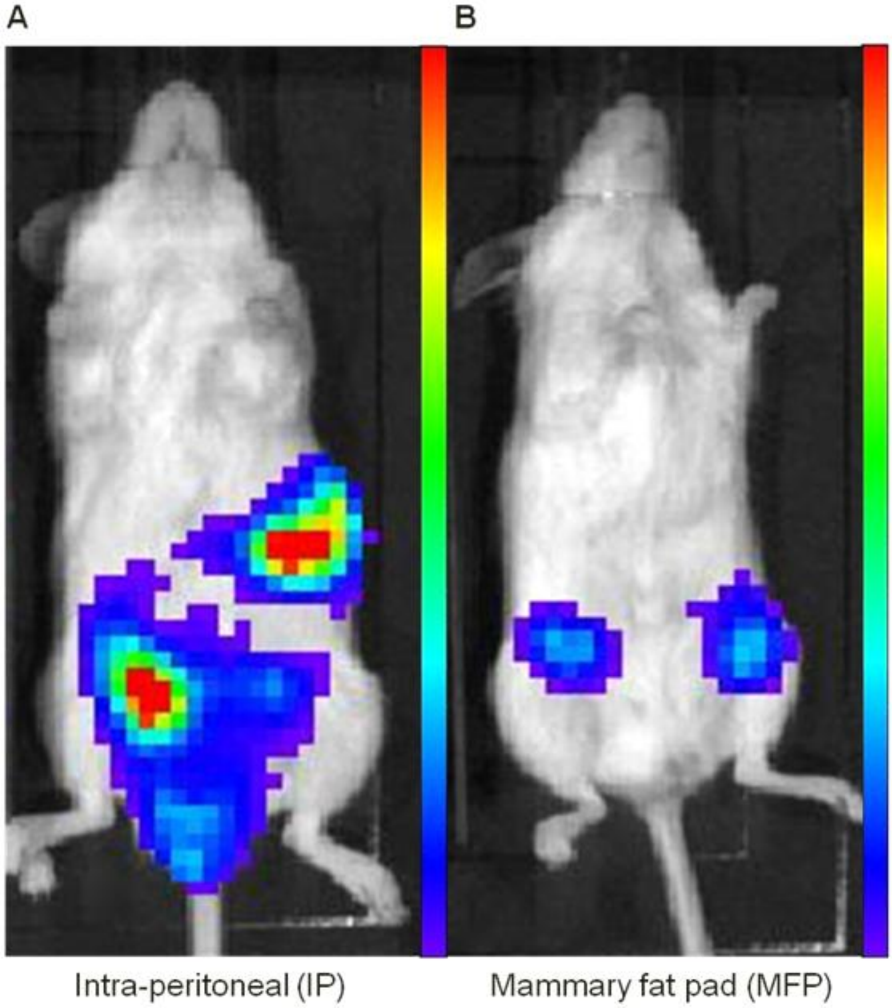

Figure S3. *In vivo* IVIS optical imaging of MDA-MB-435-MLE tumour-bearing NSG mice in different models. NSG mice were inoculated with MDA-MB-435-MLE cells intra-peritoneally or subcutaneously to produce **(A)** intraperitoneal (IP) or **(B)** mammary fat pad (MFP) tumour bearing mouse model, respectively. Bioluminescence images of mice were captured every 7 days, from day 7 post-inoculation using IVIS Lumina series III *In Vivo* Imaging Device (IVIS). In each imaging session, a total of 150 mg of luciferin per kg body weight was administered *via* subcutaneous injection.

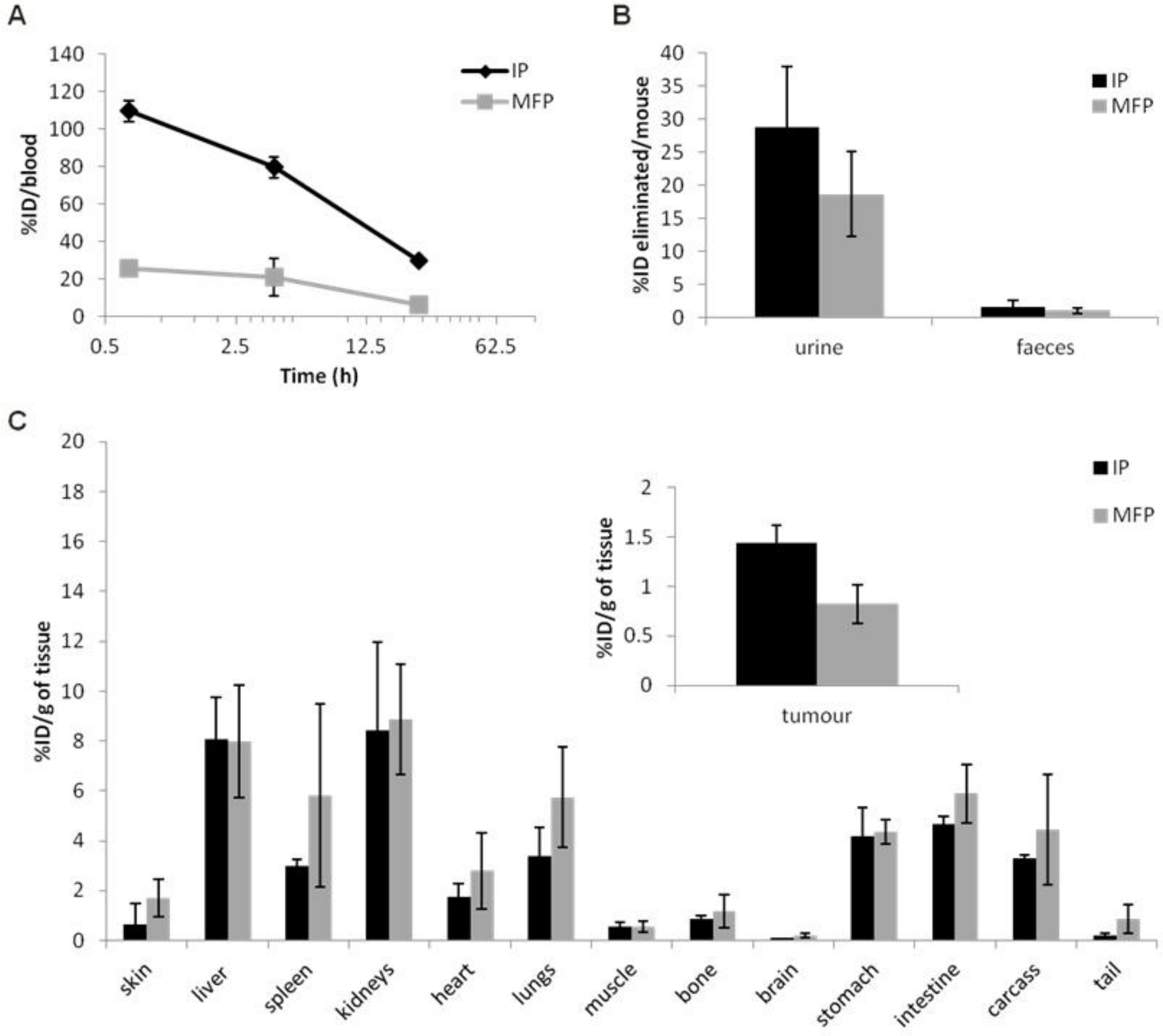

Figure S4. *In vivo* blood circulation, excretion profiles and organ biodistribution of systemically-administered [$^{99m}Tc(CO)_3]^+$ in MDA-MB-435-MLE IP or MFP tumour-bearing NSG mice. Mice were intravenously injected with [$^{99m}Tc(CO)_3]^+$ in the IP (black) and MFP (grey) tumour models, at a dose of 6-8 MBq/mouse. **(A)** Blood clearance profiles. Blood samples (5 µl) were collected from the tail vein at 30 min, 4 h and 24 h post-injection. **(B)** Excretion profile at 24 h post injection. (C) Organ biodistribution profile with values expressed as %ID/g of tissue; Inset shows the %ID/g tumour uptake. Results are expressed as mean ± SD (n=3).

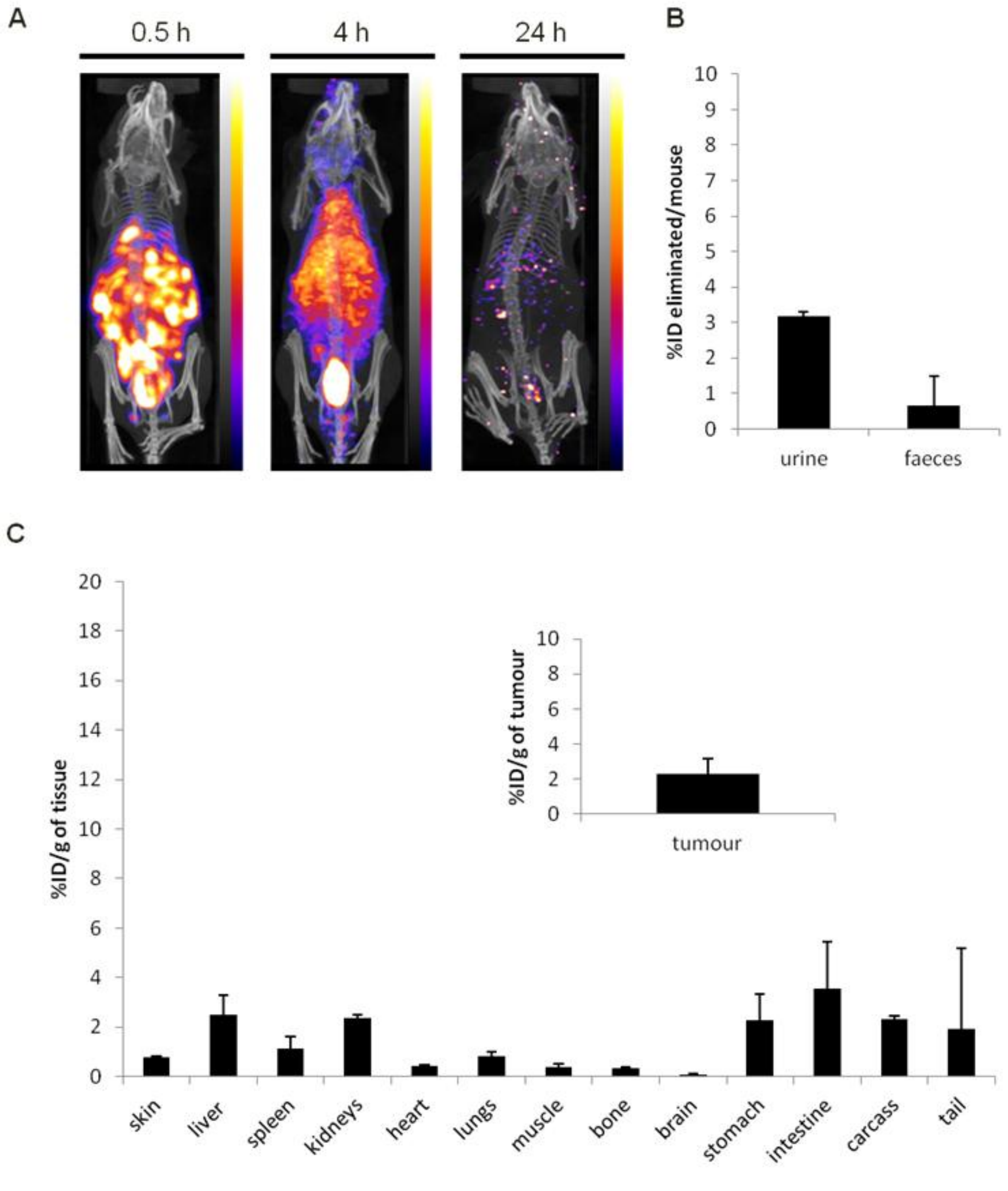

Figure S5. *In vivo* SPECT/CT imaging, excretion profile and organ biodistribution studies of locally-administered [^{99m}Tc(CO)$_3$]$^+$ in MDA-MB-435-MLE IP tumour-bearing NSG mice. Mice were intraperitoneally injected at a dose of 6-8 MBq/mouse. Organs were excised at 24 h post-injection for gamma counting. **(A)** Whole body SPECT/CT imaging at 0-30 min, 4 and 24 h post-injection of [^{99m}Tc(CO)$_3$]$^+$. **(B)** Excretion profile at 24 h post-injection, expressed as mean of %ID eliminated/mouse ± SD (n=3). (C) Organ biodistribution profile with values expressed as %ID/g of tissue; Inset shows the %ID/g tumour uptake. Results are expressed as mean ± SD (n=3).

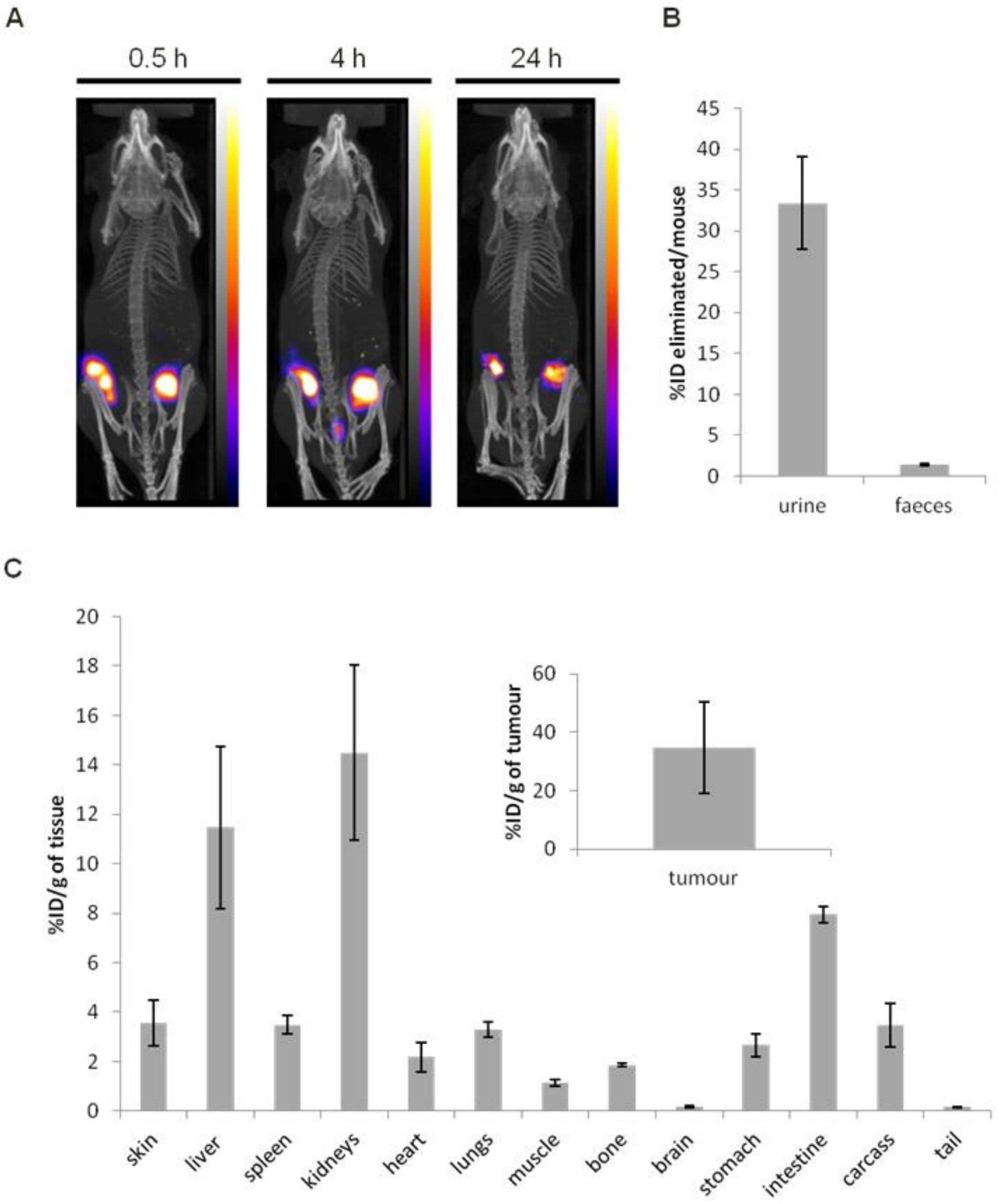

Figure S6. ***In vivo*** **SPECT/CT imaging, excretion profile and organ biodistribution studies of locally-administered [^{99m}Tc(CO)$_3$]$^+$ in MDA-MB-435-MLE MFP tumour-bearing NSG mice.** Mice were intratumourally injected at a dose of 6-8 MBq/mouse. Organs were excised at 24 h post-injection for gamma counting. **(A)** Whole body SPECT/CT imaging at 0-30 min, 4 and 24 h post-injection of [^{99m}Tc(CO)$_3$]$^+$. **(B)** Excretion profile at 24 h post injection, expressed as mean of %ID eliminated/mouse ± SD (n=3). (C) Organ biodistribution profile with values expressed as %ID/g of tissue; Inset shows the %ID/g tumour uptake. Results are expressed as mean ± SD (n=3).

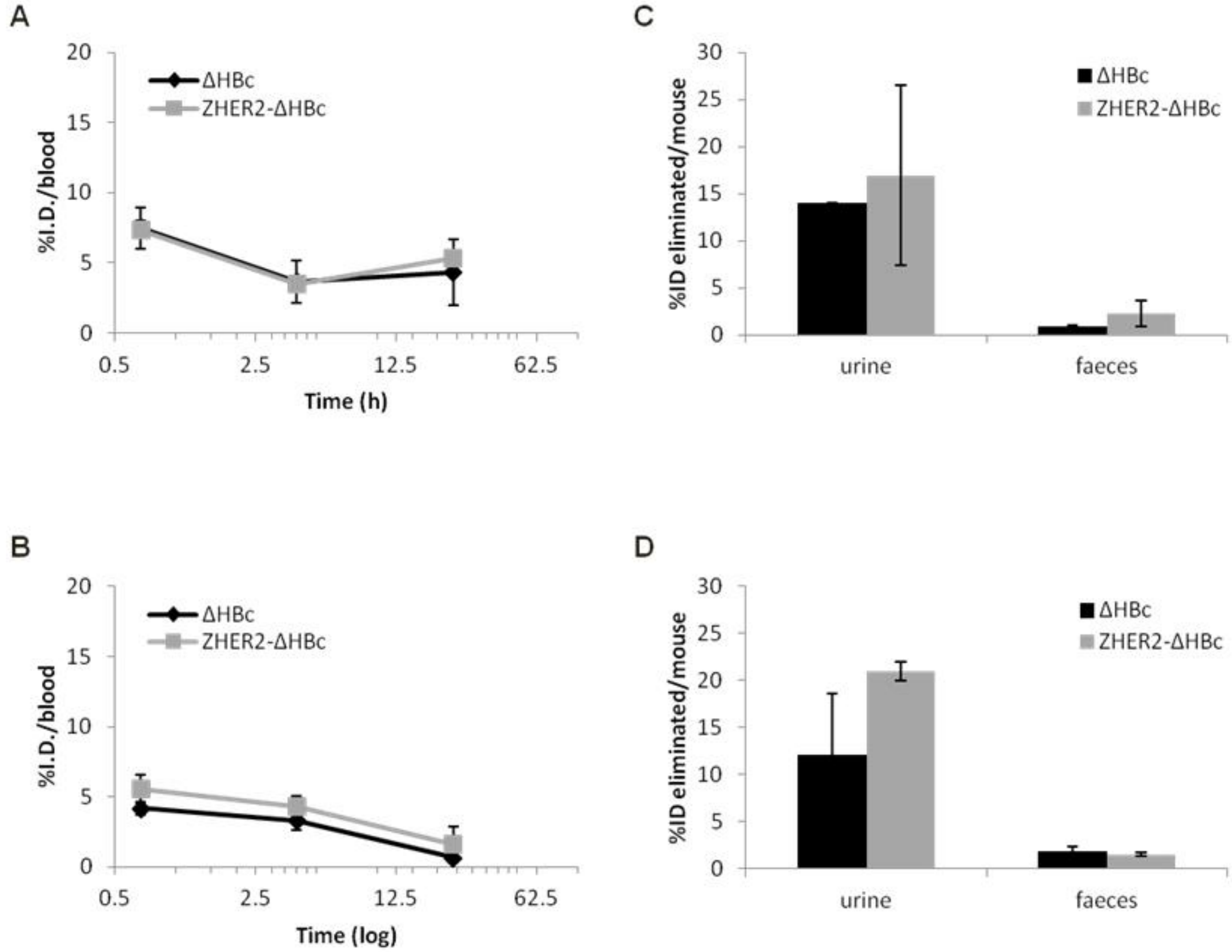

Figure S7. ***In vivo*** **blood circulation and excretion profiles of systemically-administered ^{99m}Tc-HBc particles in IP or MFP model MDA-MB-435-MLE tumour-bearing NSG mice.** Mice were intravenously injected with ^{99m}Tc-ΔHBc (black) or ^{99m}Tc-Z_{HER2}-ΔHBc (grey), in the IP (A, C) or MFP (B, D) tumour mouse model, at a dose of 50 µg protein/mouse (4-6 MBq per mouse). **(A, B)** Blood clearance profiles and **(C, D)** Excretion profile at 24 h post injection. Blood samples (5 µl) were collected from the tail vein at 30 min, 4 h and 24 h post-injection. Low detection of ^{99m}Tc-ΔHBc or ^{99m}Tc-Z_{HER2}-ΔHBc (2.5-3.0%) was detected in blood at 30 min post-injection or both models. Results are expressed as mean ± SD (n=3).

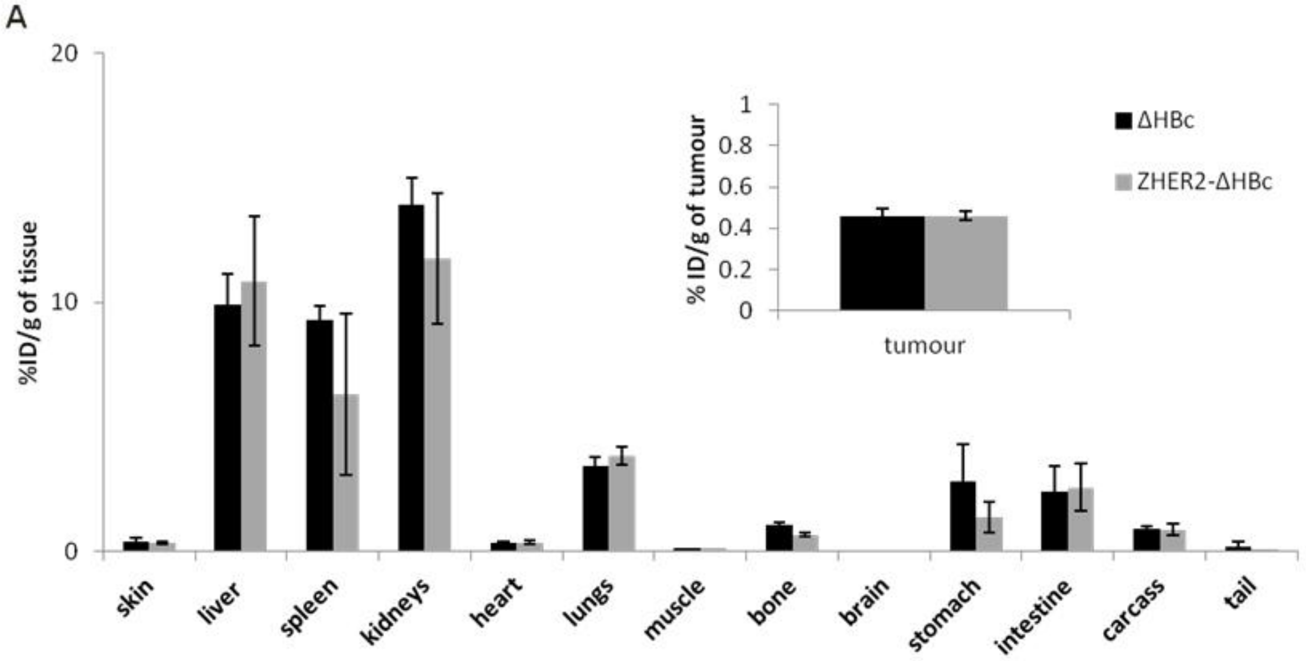

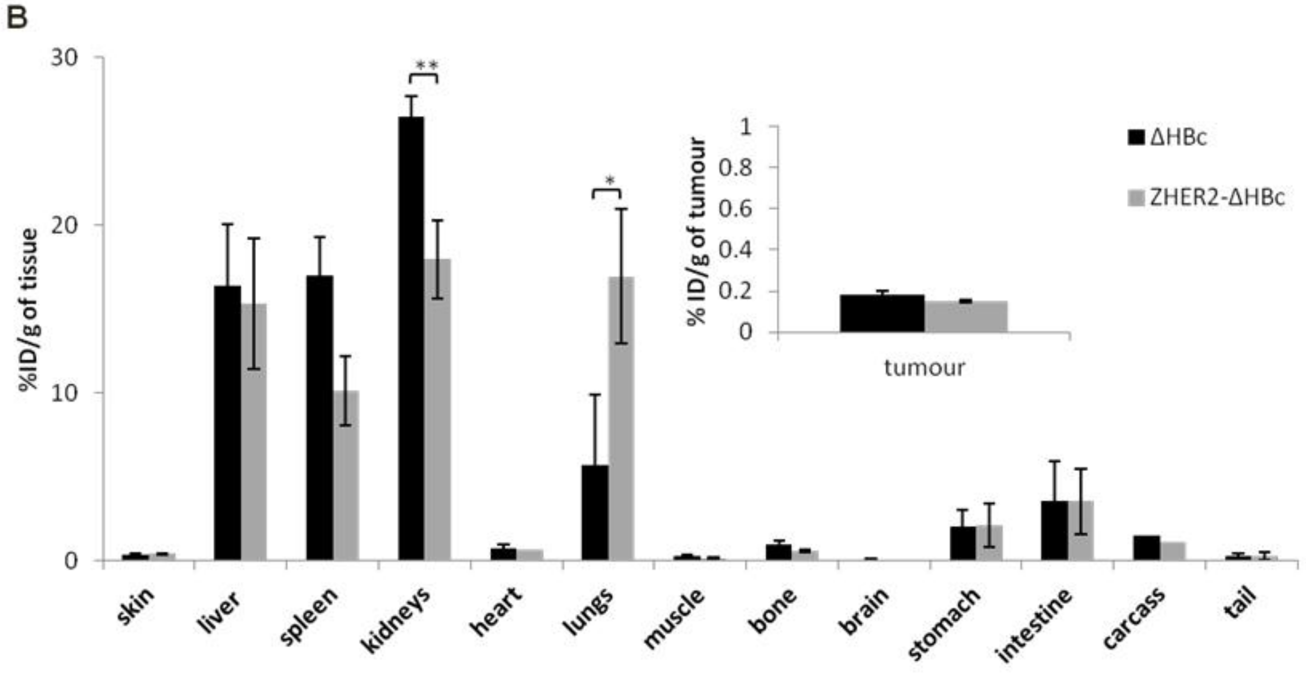

Figure S8. ***In vivo*** **organ biodistribution studies of systemically-administered ^{99m}Tc-HBc particles in IP or MFP MDA-MB-435-MLE tumour-bearing NSG mice.** Mice were injected with ^{99m}Tc-ΔHBc (black bars) or ^{99m}Tc-Z_{HER2}-ΔHBc (grey bars), intravenously in the **(A)** IP or **(B)** MFP tumour mouse model, at a dose of 50 µg protein/mouse (4-6 MBq per mouse). Organs were excised at 24 h post-injection for gamma counting. Results are expressed as mean of %ID/g of tissue ± SD (n=3).

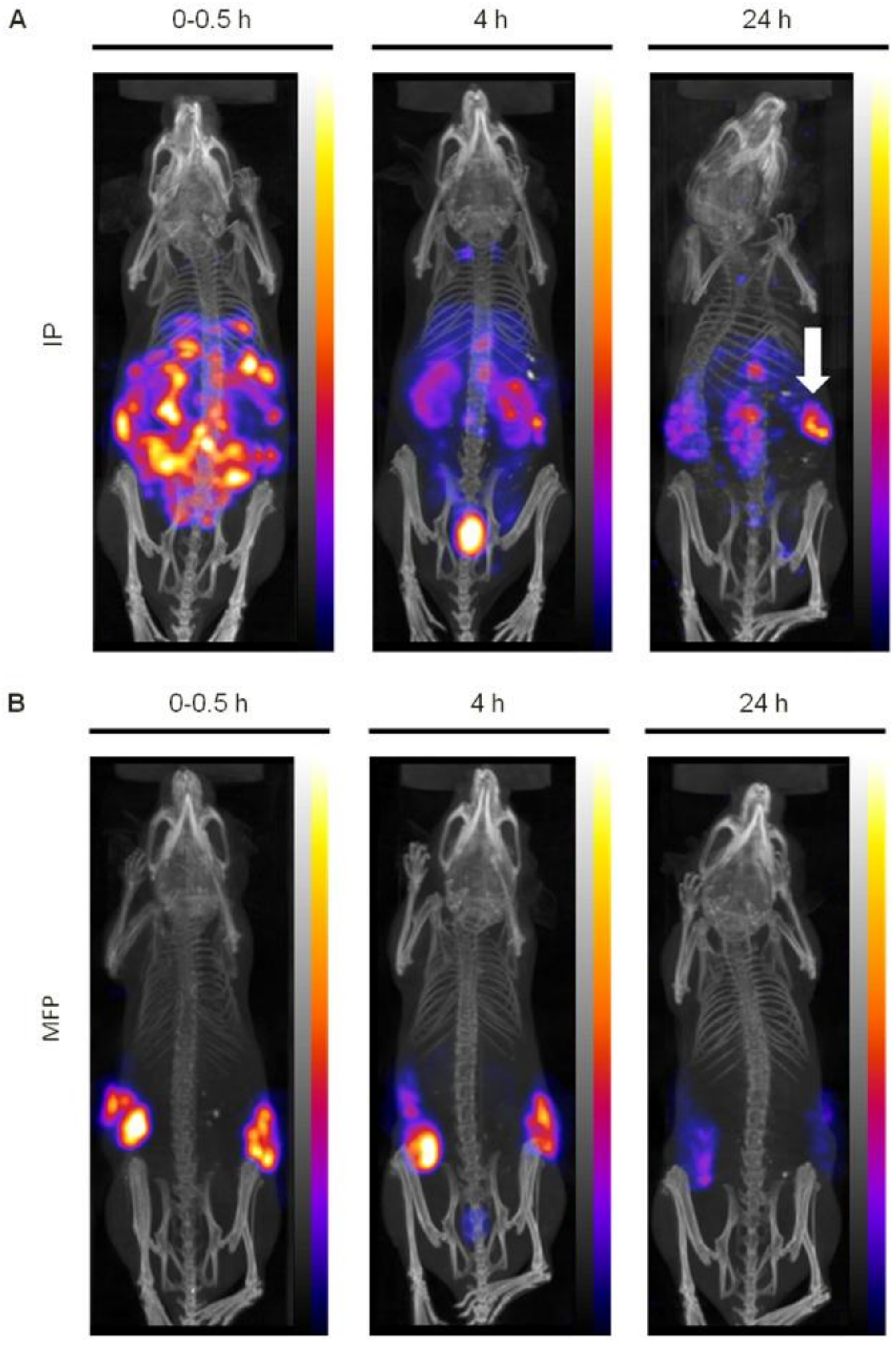

Figure S9. ***In vivo*** **SPECT/CT imaging of ^{99m}Tc-ΔHBc particles in MDA-MB-435-MLE IP and MFP tumour-bearing NSG mice.** Mice were injected with ^{99m}Tc-ΔHBc, intra-peritoneally, at a dose of 200 µg protein/mouse (6-14 MBq per mouse) in **(A)** IP or **(B)** MFP tumour-bearing mice model. Whole body 3D SPECT/CT imaging at 0-0.5, 4 and 24 h post-injection with a scanning time of 30 min each. White arrow indicates accumulation at tumour region at 24 h post-injection.

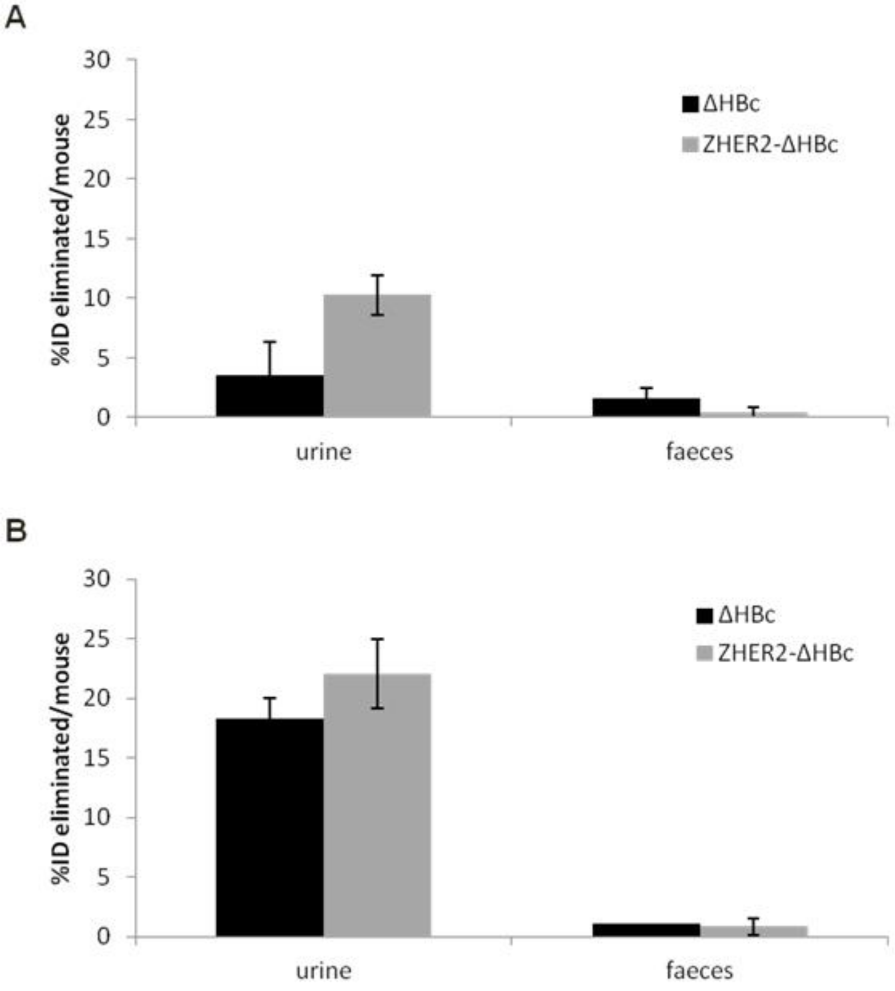

Figure S10. ***In vivo*** **excretion profile of locally-administered [^{99m}Tc(CO)$_3$]$^+$ in MDA-MB-435-MLE IP or MFP tumour-bearing NSG mice.** Mice were injected with ^{99m}Tc-ΔHBc (black bars) or ^{99m}Tc-Z_{HER2}-ΔHBc (grey bars), intraperitoneally or intratumourally in the **(A)** IP or **(B)** MFP mouse tumour model, respectively, at a dose of 50 µg protein/mouse (4-6 MBq per mouse). Excretion profile at 24 h post injection is expressed as mean of %ID eliminated/mouse ± SD (n=3).
